# Reconfiguration of large‐scale functional connectivity in patients with disorders of consciousness

**DOI:** 10.1002/brb3.1476

**Published:** 2019-11-26

**Authors:** Darwin E. Martínez, Jorge Rudas, Athena Demertzi, Vanessa Charland‐Verville, Andrea Soddu, Steven Laureys, Francisco Gómez

**Affiliations:** ^1^ Department of Systems and Computing Engineering Facultad de Ingeniería Universidad Nacional de Colombia Bogotá Colombia; ^2^ Department of Systems Engineering Universidad Central Bogotá Colombia; ^3^ Department of Biotechnology Universidad Nacional de Colombia Bogotá Colombia; ^4^ Coma Science Group GIGA‐Research and Cyclotron Research Centre University of Liège Liège Belgium; ^5^ Physics and Astronomy Department Brain and Mind Institute Western University London ON Canada; ^6^ Departamento de Matemáticas Facultad de Ciencias Universidad Nacional de Colombia Bogotá Colombia

**Keywords:** disorders of consciousness, functional connectivity, integration, segregation and centrality network measurements

## Abstract

**Introduction:**

Functional connectivity alterations within individual resting state networks (RSNs) are linked to disorders of consciousness (DOC). If these alterations influence the interaction quality with other RNSs, then, brain alterations in patients with DOC would be characterized by connectivity changes in the large‐scale model composed of RSNs. How are functional interactions between RSNs influenced by internal alterations of individual RSNs? Do the functional alterations induced by DOC change some key properties of the large‐scale network, which have been suggested to be critical for the consciousness emergence? Here, we use network analysis to measure functional connectivity in patients with DOC and address these questions. We hypothesized that network properties provide descriptions of brain functional reconfiguration associated with consciousness alterations.

**Methods:**

We apply nodal and global network measurements to study the reconfiguration linked with the disease severity. We study changes in integration, segregation, and centrality properties of the functional connectivity between the RSNs in subjects with different levels of consciousness.

**Results:**

Our analysis indicates that nodal measurements are more sensitive to disease severity than global measurements, particularly, for functional connectivity of sensory and cognitively related RSNs.

**Conclusion:**

The network property alterations of functional connectivity in different consciousness levels suggest a whole‐brain topological reorganization of the large‐scale functional connectivity in patients with DOC.

## Graphical abstract

Functional connectivity alterations within individual resting state networks are linked to disorders of consciousness. Specifically, connectivity variations indicate better integration and segregation for healthy subjects than for patients, while centrality changes suggest a role alteration in sensorimotor and auditory networks for patients.

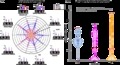

## 1 INTRODUCTION

Disorders of consciousness (DOC) encompass a set of particular conditions occurring after coma (Bruno, Laureys, & Demertzi, 2013), including the minimally conscious state (MCS), in which patients exhibit signs of fluctuating, yet, reproducible remnants of nonreflex behavior, and the unresponsive wakefulness syndrome (UWS), related to patients who open their eyes, but remain unresponsive to external stimuli (Laureys & Schiff, 2012; Schnakers & Laureys, 2012). Due to the difficult communication imposed by these conditions, brain activity registered at rest (Biswal, 2012) is used to develop complementary diagnosis approaches. In particular, resting state functional magnetic resonance imaging (R‐fMRI) protocols are used to understand brain activity while subjects are not exposed to stimuli (Demertzi et al., 2014; Sporns, 2013), overcoming the need for their active participation. R‐fMRI studies in healthy controls (HC) suggest that the brain is organized into large‐scale resting state networks (RSNs) of sensory/cognitive relevance (Fox & Raichle, 2007; Rosazza & Minati, 2011). At least ten of these functional entities were identified in HC including auditory, cerebellum, default mode network (DMN), executive control left (ECN Left), executive control right (ECN Right), saliency, sensorimotor, visual lateral, visual media, and visual occipital (Damoiseaux et al., 2006). RSNs provide a suitable representation to study the preservation of sensorial and cognitive brain functions without any explicit stimulation (Rosazza & Minati, 2011) specifically for DOC studies.

First analyses of RSNs in patients with DOC focused on alterations of the functional connectivity inside the DMN. This is a functional structure that encompasses specific brain regions linked to the consciousness emergence phenomenon (Boly et al., 2008; Demertzi, Soddu, & Laureys, 2013). Decreases in functional connectivity within this network are linked to modifications of the level of consciousness in these patients. Posterior studies showed that DOC conditions may affect functional connectivity within multiple RSNs (Demertzi et al., 2014, 2013; Di Perri, Stender, Laureys, & Gosseries, 2014; Di Perri, Thibaut, et al., 2014; Guldenmund et al., 2013; Heine et al., 2012; Ribeiro de Paula et al., 2017). In particular, variations in intrinsic connectivity for specific RSNs were related to alterations in sensorial and awareness functions (Boly et al., 2008; Demertzi et al., 2013; Di Perri, Stender, et al., 2014). Additional evidence indicates changes in the connectivity between RSNs, for instance, reductions of the connectivity strength between RSNs in patients with DOC compared to HC subjects (Rudas et al., 2014) and alterations in the level of anti‐correlation between RSNs associated with the recovery of consciousness (Di Perri et al., 2016). In summary, these analyses focused on alterations within particular RSNs or between specific pairs of RSNs that may have functional relevance for consciousness emergence.

Nevertheless, these approaches may be limited because they do not consider a more general view of the brain, regarding, for instance, the existence of multiple functional units in the brain and the interactions among them (van den Heuvel & Hulshoff Pol, 2010). They are instead focused on specific consciousness‐related circuits within the brain. A more general perspective is important because consciousness preservation in these patients would also require functional units related not only to consciousness processing but also to stimuli and response, and possibly systems to orchestrate them (Tononi & Koch, 2015). The understanding of interactions among these units may provide valuable information about these conditions (Tononi & Koch, 2015). Recently, a model of functional connectivity among RSNs has been proposed in the so‐called functional network connectivity (FNC; Jafri, Pearlson, Stevens, & Calhoun, 2008), which considers the functional interaction between these large‐scale units. This model provides a network representation in which interactions between high‐order functional systems can be characterized using network measurements (Bullmore & Sporns, 2009, 2012; van den Heuvel & Hulshoff Pol, 2010). Lately, connectivity density decreases were associated with consciousness alterations in coma, providing a general description of FNC alterations (Malagurski et al., 2019). However, the specific reconfiguration of FNC associated with consciousness states is not tackled.

In this study, we hypothesize that the FNC model may highlight reorganizations of connectivity related to the underlying pathology characterizing the DOC condition. These interaction patterns were studied by assessing modifications in integration, segregation, and centrality properties, which have been suggested to be highly relevant for consciousness emergence (Tononi & Koch, 2015). These properties were analyzed for three populations in different states of consciousness: healthy controls, subjects with MCS, and subjects with UWS. In contrast to previous studies that only focused on a limited set of RSNs, we considered the interactions among the whole set of functional units. To reach this objective, the FNC was computed for each subject obtaining a general brain functional representation with the interactions between RSNs. Next, we used a set of network measurements to assess the mentioned properties. In particular, degree, strength, clustering coefficient, betweenness, and eigenvector centralities were used to understand key brain functional property modifications for different states of consciousness. Degree and strength assess the integration between functional brain regions, that is, how the regions are connected and how strong are the connections, respectively. Clustering coefficient measures the segregation of brain regions, that is, how the regions are interconnected creating functional units. Betweenness and eigenvector centralities evaluate the relevance of a region in the functional model, that is, how important a region is for the communication because it belongs to the shortest path or it is connected to other relevant regions, respectively. Our results suggest that decreases in the level of consciousness in patients with DOC are related with a topological reorganization of large spatial scale connectivity, involving not only regions directly related to consciousness, as DMN, but also other functional systems of sensorial and cognitive relevance. This finding has a major implication in functional studies related to consciousness, suggesting a reconfiguration of sensorial and cognitive systems, in particular, reconfigurations that may involve brain adaptations due to communication impairment.

## 2 MATERIALS AND METHODS

**Figure 1:**
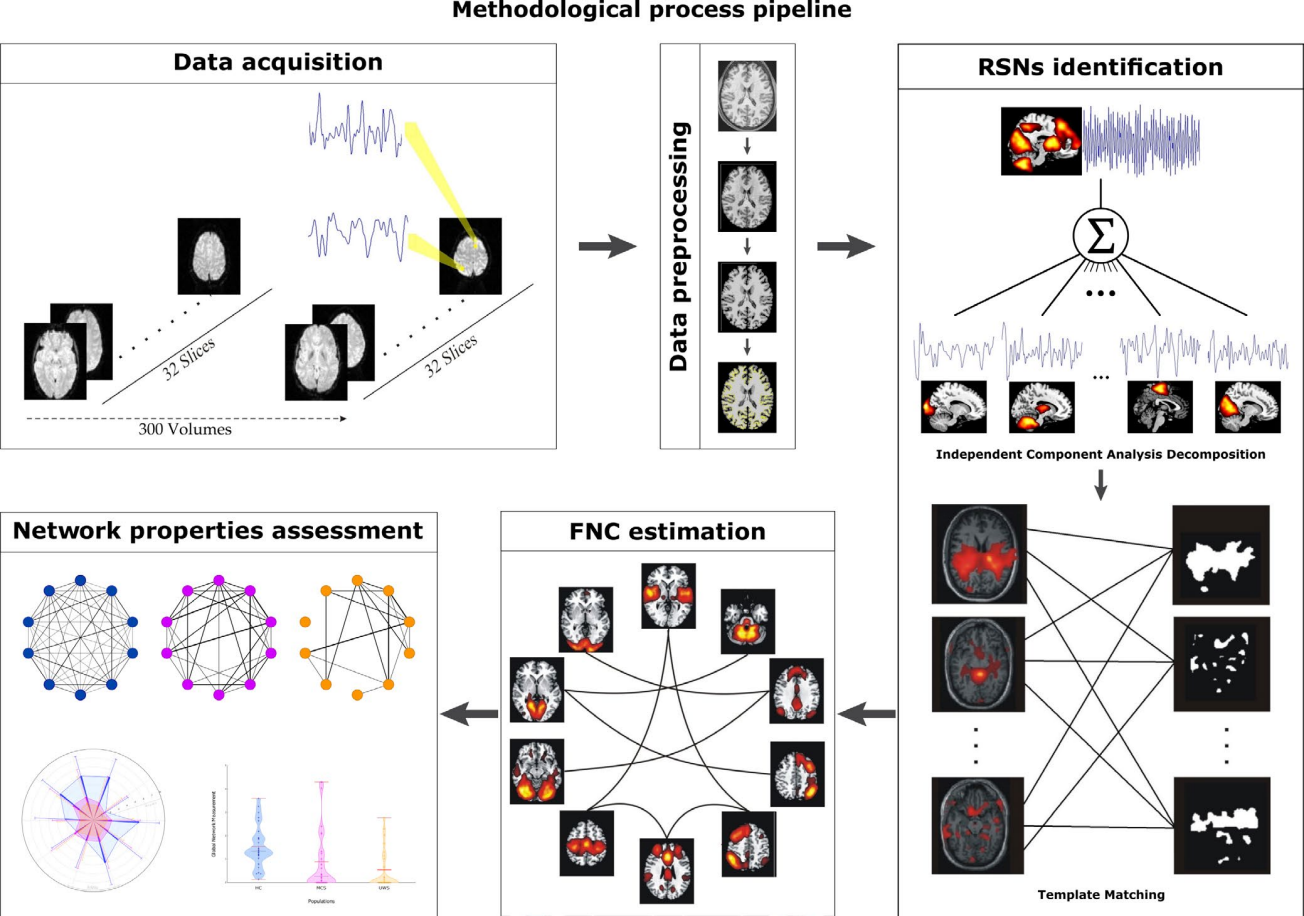
Illustration of the methodological procedure defined as the sequence of the following processes: data acquisition consisting of 300 volumes of functional magnetic resonance imaging (MRI) at rest and a structural MRI for each subject; data preprocessing including brain extraction, alignment, registration, Gaussian smoothing, motion correction, and normalization; extraction of the resting state networks (RSNs) using spatial independent component analysis and a template matching strategy under a data‐driven approach; computation of functional network connectivity (FNC) between RSNs by the lagged distance correlation method; and finally, computation of integration, segregation and centrality measurements to characterize the populations in different states of consciousness

We aim to characterize a functional connectome for HC subjects and patients with DOC (Bullmore, 2012; Calhoun, Adali, & Pearlson, 2001; Sporns, 2013; van den Heuvel & Hulshoff Pol, 2010), in particular, a connectome with RSNs as nodes. This connectome corresponds to a large‐scale network of functional relationships between functionally related brain regions. Network‐based measurements computed on this connectome provide a functional depiction of synchronized, spontaneous, and segregated activity (Rubinov & Bullmore, 2013; Sporns, 2013; van den Heuvel & Hulshoff Pol, 2010). Importantly, there is a methodological challenge in the characterization of the functional relationship between large‐scale areas (RSNs) in patients with severe brain damage. Particularly, brain‐injured patients may present functional and structural affectations that may change the connectome properties. Therefore, in this study, a particular processing pipeline that accounts for these alterations were considered, including, severe structural affectations, large head motions, and individual variability, among others. Figure 1 summarizes the process used to characterize functional connectivity alterations at the general brain level of interactions between RSNs.

### 2.1 Subjects and patients

Participants were healthy volunteers and patients with UWS or MCS following severe brain damage studied at least 5 days after acute brain insult. HC subjects were subjects free of psychiatric or neurological history. Clinical examination was performed using the French version of the Coma Recovery Scale‐Revised (CRS‐R; Giacino, Kalmar, & Whyte, 2004; Schnakers et al., 2008). The CRS‐R is a standardized measure for characterizing the level of consciousness and monitoring recovery of neurobehavioral function (Giacino et al., 2004). It consists of 30 hierarchically arranged items that comprise six subscales addressing auditory (5 items), visual (6 items), motor (7 items), oromotor/verbal (4 items), communication (4 items), and arousal (4 items) processes. The scoring is based on the presence or absence of specific behavioral responses to sensory stimuli administered in a standardized manner, and the lowest item in each subscale represents reflexive activity while the highest item represents cognitively mediated behaviors (Giacino et al., 2004; Schnakers et al., 2008). Exclusion criteria were contraindication for MRI (e.g., presence of ferromagnetic aneurysm clips, pacemakers), MRI acquisition under sedation or anesthesia and large focal brain damage (>50% of total brain volume). Structural brain damage was assessed by visual inspection of two experts. Written informed consent to participate in the study was obtained from the healthy subjects and from the legal surrogates of the patients. The study was approved by the Ethics Committee of the Medical School of the University of Liège (Demertzi et al., 2014).

### 2.2 Data description

Acquisitions from 75 subjects were used for this study: 27 HC subjects (14 women, mean age 47 ± 16 years), 24 patients with MCS (eight women, mean age 47 ± 16 years; nine of nontraumatic etiology: two anoxic, three with cerebrovascular accident, three with hemorrhage, one with seizure; 14 of traumatic, and one of mixed etiology), and 24 with UWS (12 women, mean age 50 ± 18 years; 18 of nontraumatic etiology: nine anoxic, six with cerebrovascular accident, two with hemorrhage, one metabolic; five of traumatic, and one of mixed etiology). Thirty‐one patients with UWS and MCS were assessed in the chronic setting, that is, ≥50 days postinsult. Further details about the patients' demography can be found in Table S1.

For each subject, fMRI data were acquired in a 3T scanner (Siemens Medical Solution). Three hundred fMRI volumes multislice T2*‐weighted functional images were captured (32 slices; voxel size: 3 × 3 × 3 mm^3^; matrix size 64 × 64 × 32; repetition time = 2,000 ms; echo time = 30 ms; flip angle = 78°; field of view = 192 × 192 mm^2^). The three initial volumes were discarded to avoid T1 saturation effects. In addition, for anatomical reference, a structural T1‐weighted image was acquired. Patients were scanned in sedation‐free condition, and healthy volunteers were instructed to close their eyes, relax without falling asleep and refrain from any structured thinking (e.g., counting and singing), as commonly performed in resting state paradigms (Beckmann, Luca, & Devlin, 2005; Guldenmund et al., 2013).

### 2.3 Data preprocessing

Data preprocessing was performed using the Statistical Parametric Mapping (SPM8) (Friston, 2007) toolbox for Matlab (The Mathworks, Inc.). SPM preprocessing stages included realignment and adjustment for movement‐related effects, coregistration of functional onto structural data, segmentation of structural data, normalization into standard stereotactic MNI space, and spatial smoothing with a Gaussian kernel of 8 mm. To evaluate the data acquisition quality, the frame‐wise displacement (Power, Barnes, Snyder, Schlaggar, & Petersen, 2012) was assessed on each population, further details Supplementary Material Section 2. Motion correction (e.g., small, large and rapid motions, noise spikes and spontaneous deep breaths) was applied by using ArtRepair toolbox for SPM (Demertzi et al., 2014; Mazaika, Hoeft, Glover, & Reiss, 2009).

### 2.4 Resting state networks identification

For each subject, the resting state networks (RSNs) were selected as follows: First, the rs‐fMRI signal was decomposed into maximally independent spatial maps using spatial ICA (McKeown et al., 1998). ICA decomposition was performed with 30 components (Jafri et al., 2008) and the infomax algorithm as implemented in GroupICA toolbox (Calhoun et al., 2001). Each spatial map (source fMRI signal) has an associated time‐course, which corresponds to the common dynamic exhibit by the component. Second, RSNs were identified at individual level (Demertzi et al., 2014) by using a two‐fold process: template matching and neuronal/artifactual classification (see Supporting Information Section 4). Template Matching is an approach that aims to identify each RSN directly from the single subject sICA decomposition (Demertzi et al., 2014). It is a matching problem with two constraints: (a) a template had to be assigned to one of the 30 ICs and (b) an IC could be labeled as an RSN or not. These two conditions ensure that all the templates (one for each RSN) have to be assigned and a unique identification of each IC, which deal with the potential concurrent component assignation. The pair between the template and the IC with the highest goodness‐of‐fit score was selected (Demertzi et al., 2014). Later, a neuronal/artifactual classification of independent components (ICs) was performed by using a machine learning‐based labeling method (Demertzi et al., 2014). It consists of a binary classification approach by means of support vector machine (SVM) classifier trained on 19 independently assessed healthy subjects. This SVM uses the fingerprints obtained from ICA decomposition (*n* = 30 components) as the feature vector containing both spatial (i.e., degree of clustering, skewness, kurtosis, spatial entropy) and temporal information (i.e., one‐lag autocorrelation, temporal entropy, power of five frequency bands: 0–0.008 Hz, 0.008–0.02 Hz, 0.02–0.05 Hz, 0.05–0.1 Hz, and 0.1–0.25 Hz). Commonly, components of artifactual origin encompasses (a) high‐frequency fluctuations >0.1Hz, (b) spikes, one or more abrupt changes in the normalized time‐course, (c) the presence of sawtooth pattern, and (d) the presence of threshold voxels in the superior sagittal sinus. Finally, neuronal time‐courses of the RSNs were extracted at the individual level, and they were subsequently used for the functional connectivity computations.

### 2.5 Functional network connectivity estimation

For each subject, a FNC matrix was computed by using a measure of dependency between pairs of representative time‐courses, resulting in a matrix with strengths of the interactions between the identified RSNs. The strength for edges pointing to RSNs marked as no‐neuronal was set as zero, indicating no interaction. The measures of dependency level were computed using the distance correlation (DC) between time‐courses (Székely, Rizzo, & Bakirov, 2007). DC aims to measure nonlinear dependencies between two random variables X and Y with finite moments in arbitrary dimension. In order to account for time delays, a circular shifted lagged version of the DC was used (Jafri et al., 2008; Rudas et al., 2014). Once the FNC was computed, it induces a functional connectivity matrix, which was used to characterize alterations of functional connectivity. In particular, a 10 × 10 weighted matrix was computed to model interactions between different RSNs. Each one of them models a brain region associated with specific arousal and awareness regions related to consciousness emergence. An entry *c_ij_* in this matrix corresponds to the interaction between the RSN*_i_* and RSN*_j_* assessed by using the lagged DC. Further details of this approach can be found in Supporting Information Section 5.

### 2.6 Network characterization

Functional network connectivity matrix contains a measure of dependency between pairs of RSNs time‐courses. To assess functional connectivity alterations, three network properties were computed for each FNC matrix, namely, integration, segregation and centrality of the functional connectivity between RSNs. FNC integration was assessed by degree and strength (Bullmore & Sporns, 2009, 2012; Rubinov & Sporns, 2010). FNC segregation was characterized by clustering coefficient (Bullmore & Sporns, 2009, 2012; Rubinov & Sporns, 2010), and FNC centrality was estimated by betweenness centrality and eigenvector centrality (Lohmann et al., 2010; see a brief description of the network measurements in Table S3). These computations were performed using the brain connectivity toolbox (Rubinov & Sporns, 2010).

Functional network connectivity degree values quantify the number of nonzero correlations of each RSN with other nodes in the network, while strength values indicate not only a correlation between RSNs but also the robustness of this correlation. They also provide a measure of the communication quality expressed in the correlation, that is, higher values for these two measurements indicate better communication. Similarly, FNC segregation was measured by clustering coefficient. This assessment indicates how well‐connected neighbor nodes are in order to become a grouped unit. High clustering coefficient values indicate that a set of nodes are well connected among themselves. Additionally, FNC centrality was assessed by betweenness and eigenvector measurements. Higher betweenness centrality values of a RSN mean that a node belongs to a high number of the shortest paths (path with the minimum distance between two nodes) between pairs of nodes in the network. For example, when a RSN time‐course is better related to other time‐course in sequence, it presents a better communication path. Furthermore, a higher RSN eigenvector centrality value indicates that this RSN is better connected to other central nodes. This estimates how central a RSN is based on the direct connections to others that have strong links. All measurements herein used were computed for each node, that is, for each RSN in the FNC. Average measurements were calculated to quantify communication quality among the network nodes. They describe the global network functional connectivity properties and depict all the network variations associated with FNC alterations.

### 2.7 Statistical analysis

To assess the discrimination power of the network properties, an unpaired‐sample *t* test (Welch, 1947; Bonferroni corrected) was computed. For the statistical analysis, the following comparisons were performed: HC versus subjects with MCS, HC versus subjects with UWS, HC versus subjects with DOC (UWS and MCS), and subjects with MCS versus subjects with UWS.

## 3 RESULTS

In this study, a set of network measurements were used to assess the integration, segregation, and centrality of the FNC between RSNs to characterize connectivity variations in different states of consciousness.

### 3.1 Loss of functional network connectivity integration in DOC

**Figure 2:**
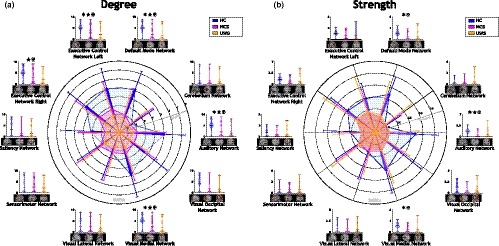
Integration measurements. (a) Degree and (b) strength show a similar distribution across healthy subjects and patients with disorders of consciousness (DOC). Both evidence higher values for healthy controls (HC) than subjects with DOC in the same resting state networks (RSNs; auditory, cerebellum, default mode network [DMN], executive control network [ECN] Left, ECN Right, saliency, sensorimotor, visual lateral, visual media and visual occipital). Significant differences between HC and minimally conscious state (MCS) and unresponsive wakefulness syndrome (UWS) patients were assessed in RSNs associated with the phenomenon of consciousness emergence (auditory, DMN, ECN Left, ECN right, Saliency). Fingerprints lines indicate mean values, and thin lines indicate standard deviation values for each RSN. ✶ aims for significant difference between HC and MCS. ★ aims for significant difference between HC and UWS. ✠ aims for significant difference between HC and DOC

Figure 2 shows degree and strength values for subjects in different states of consciousness for the 10 different RSNs herein studied. As observed in Figure 2a, degree values were higher for HC compared to subjects with altered states of consciousness (MCS and UWS) in all RSNs, except by the sensorimotor network. Significant differences (*p* < .005) were observed for the values of degree when comparing HC with MCS populations in auditory network, DMN, ECN Left and visual medial network. Significant differences (*p* < .005) were also found when comparing HC versus subjects with UWS and when comparing HC and subjects with DOC in auditory network, DMN, ECN Left, visual medial network, and ECN Right. Also, degree values for subjects with MCS were greater than the UWS in all RSN but no significant differences were observed. Table S4 reports statistical details of these assessments.

As observed in Figure 2b, strength values were higher for HC in comparison to subjects with altered states of consciousness in all RSNs except by sensorimotor and cerebellum networks. Significant differences (*p* < .005) in strength values were observed for HC compared to subjects with MCS and for HC versus the population of DOC, in auditory network, DMN and visual medial network. HC presented strength values significantly higher than subjects with UWS. No significant differences were observed between strength values of subjects with MCS compared to subjects with UWS. Table S5 reports the statistical details about strength value comparisons.

**Figure 3:**
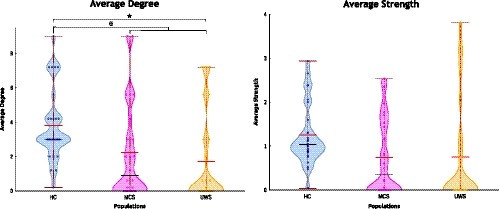
Distribution of the average integration measurements for the three populations herein studied. (a) Degree and (b) Strength. Red lines indicate the mean, black lines indicate the median and red wine lines indicate the maximum. Each dot in the violin represents the measurement on a single subject. ✶ aims for a significant difference between healthy controls (HC) and minimally conscious state (MCS; *p* < .05). ★ aims for a significant difference between HC and unresponsive wakefulness syndrome (UWS; *p* < .05). ✠ aims for a significant difference between HC and patients with disorders of consciousness *p* < .05)

Figure 3 shows the average degree and average strength values. Average values were estimated as a global characteristic of functional connectivity network between RSNs. As observed in Figure 3a, average degree values were higher for HC compared to altered states of consciousness. Significant differences were also found for HC (*M* = 3.81, *SD* = 2.10) when compared with UWS (*M* = 1.71, *SD* = 2.59; *t* = 3.16, *p* = .003). Similarly, significant differences in average degree were also found when HC subjects were compared with DOC (*M* = 1.97, *SD* = 2.69; *t* = 3.03, *p* = .003). Further, the average degree presented a decreasing tendency which corresponds to the increase in DOC severity. As observed in Figure 3b, average strength values were higher for HC compared to altered states of consciousness. No significant differences were observed for these averages when compared among populations, in contrast to the previous observation of decreases in the average degree values. Also, average degree and average strength values exhibit greater spread distributions for subjects with MCS and subjects with UWS than for HC subjects.

### 3.2 Loss of functional network connectivity segregation in DOC

**Figure 4:**
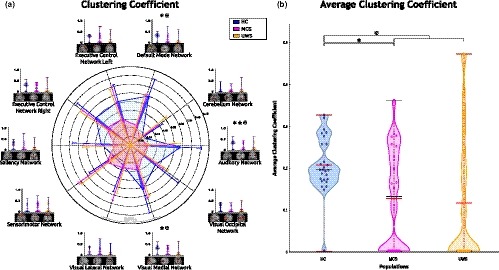
Segregation measurement between resting state networks by clustering coefficient. (a) fingerprint (b) violin plot. Higher clustering coefficient values were observed for healthy controls (HC) than for subjects with disorders of consciousness (DOC) except by sensorimotor network. ✶ aims for a significant difference between HC and patients with minimally conscious state (MCS). ★ aims for a significant difference between HC and unresponsive wakefulness syndrome (UWS). ✠ aims for a significant difference between HC and patients with DOC

Figure 4 reports the clustering coefficient values for subjects in different states of consciousness. Higher clustering coefficient values were obtained for HC in comparison to altered states of consciousness except in sensorimotor, cerebellum and visual lateral networks (Figure 4a). Clustering coefficient values for HC present significant differences (*p* < .005) compared to subjects with MCS in auditory network and DMN. Significant differences (*p* < .005) also were observed when comparing HC and subjects with UWS for auditory and visual medial networks. No significant differences of clustering coefficient values were observed for the RSN when compare subjects with UWS and subjects with MCS. Finally, differences between subjects with DOC and HC subjects were significantly distinct (*p* < .005) for auditory network, DMN and visual medial network. Table S6 reports statistical details about the comparisons performed for the clustering coefficient.

Figure 4b shows the average clustering coefficient values. These values were higher for HC compared to altered states of consciousness. Average clustering coefficient values were significantly higher for HC (*M* = 0.20, *SD* = 0.06) compared to subjects with MCS (*M* = 0.12, *SD* = 0.12; *t* = 2.95, *p* = .004). Similarly, significant differences were also higher when comparing HC and subjects with DOC (*M* = 0.12, *SD* = 0.14; *t* = 2.97, *p* = .004). Further, distributions of the average clustering coefficient were narrower for HC than for subjects with DOC while their means exhibit a slightly decreasing tendency in correspondence with the severity of DOC.

### 3.3 Alterations of functional network connectivity centrality in DOC

**Figure 5:**
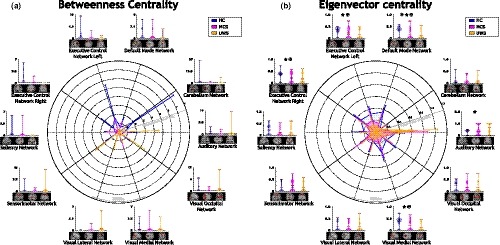
Centrality measurements. (a) Betweenness centrality exhibits a central role changing in auditory, sensorimotor, visual lateral and visual occipital networks for subjects with disorders of consciousness (DOC). Similarly, (b) eigenvector centrality presents a role alteration for auditory and sensorimotor networks in subjects with DOC. ✶ aims for a significant difference between Healthy Controls (HC) and patients with minimally conscious state (MCS). ★ aims for a significant difference between HC and patients with unresponsive wakefulness syndrome (UWS). ✠ aims for significant difference between HC and patients with DOC

Betweenness centrality and eigenvector centrality values are reported in Figure 5. Betweenness centrality values were higher for HC in contrast to subjects with altered states of consciousness for DMN, ECN Left, ECN Right, salience network and cerebellum network, as observed in Figure 5a. These centrality values of subjects with UWS were higher when comparing to subjects with MCS and when comparing to HC subjects, for auditory, sensorimotor, visual lateral, visual medial and visual occipital networks. Also, ECN Right and salience network has values of zero of betweenness centrality for UWS patients, indicating that these nodes were not part of any shortest path in the network. Table S7 reports statistical details about these comparisons.

As observed in Figure 5b, eigenvector centrality values were higher for HC compared to subjects with DOC except by auditory and sensorimotor networks. Higher values of eigenvector centrality for HC with significant differences (*p* < .005), were observed for DMN, ECN Left, ECN Right, and visual medial network compared with subjects with DOC. Similarly, when contrasting HC and subjects with MCS, significant differences (*p* < .005) were obtained for DMN. Eigenvector centrality values were significantly different (*p* < .005) for DMN, ECN Left, ECN Right and visual medial network in comparison with HC and subjects with UWS. Further, eigenvector centrality values were higher for subjects with MCS compared to HC, and for subjects with MCS versus UWS for the sensorimotor network. Finally, auditory network eigenvector centrality values were higher for subjects with UWS compared to subjects with MCS, which were also higher than HC. This observation in the auditory network indicates an increasing tendency in centrality, which corresponds with the severity of the pathology. For this network, significant differences (*p* < .005) were found between subjects with UWS and HC. Table S8 reports statistical details about eigenvector centrality comparisons.

**Figure 6:**
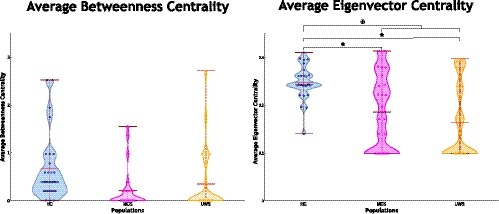
Average centrality distribution measurements (a) Betweenness Centrality, (b) Eigenvector Centrality. Red lines are the mean, black lines are the median, red wine lines are the maximum. Average eigenvector centrality shows narrower distributions for Healthy Controls (HC) than subjects with disorders of consciousness (DOC). Also, a decreasing tendency is observed in correspondence with the consciousness content. ✶ significant difference between HC and patients with Minimally Conscious State (MCS). ★ significant difference between HC and patients with Unresponsive Wakefulness Syndrome (UWS). ✠ significant difference between HC and patients with DOC

Figure 6 illustrates average betweenness centrality and average eigenvector centrality. Average betweenness centrality values were higher for HC in contrast to subjects with altered states of consciousness. Also, the distribution of these values is narrower for MCS compared to HC and subjects with UWS (Figure 6a). Similarly, higher values of average eigenvector centrality were observed for HC when comparing to subjects with DOC (Figure 6b). Significant differences were observed when compare the populations, between HC (*M* = 0.25, *SD* = 0.06) and subjects with MCS (*M* = 0.19, *SD* = 0.08; *t* = 3.61 *p* = .00071), between HC and subjects with UWS (*M* = 0.16, *SD* = 0.08; *t* = 5.04 *p* = .00001), and between HC and subjects with DOC (*M* = 0.18, *SD* = 0.08; *t* = 4.54 *p* = .00002). Further, average eigenvector centrality values exhibit a decreasing tendency as the severity of the pathology increases. They also showed a narrower distribution for HC in comparison to subjects with DOC.

## 4 DISCUSSION

In this paper, we studied whole‐brain functional connectivity changes in different states of consciousness: HC, subjects with MCS and subjects with UWS. Unlike previous approaches, which mainly focus on functional units that seem to be associated to consciousness, that is, DMN, the proposed model considers a more general set of functional units that represents connectivity between RSNs, including sensory and cognitive‐related ones. So, this model provides a general perspective of the cognitive and sensory RSNs connectivity variations in altered states of consciousness. We assessed the interactions between these RSNs, resulting in a FNC model that allows describing whole‐brain system‐level interactions. We used a model of functional connectivity among RSNs, which corresponds to large spatial scale segregated functional units. In this model, the interaction between pairs of representative time‐courses of each RSN is computed (Biswal, 2012), resulting in a FNC model that allows describing whole‐brain interactions (Jafri et al., 2008). The proposed model provides a general perspective which overcomes the specific or single consciousness‐related region studies (Demertzi et al., 2013; Di Perri, Thibaut, et al., 2014; Guldenmund et al., 2013). It allows describing the entire brain functional reconfiguration in a broad scale of regions related to sensory and cognitive processes. This reconfiguration description goes further than previous general density studies in coma (Malagurski et al., 2019) depicting the functional variations between functional units associated with the consciousness level. The proposed model differs from the previous EEG holistic functional model which is used to discriminate MCS and UWS patients using a hemispheric division of Brodmann Areas, that is, 84 regions, to build the functional connectome and a set of network measurements revealing alterations in the small‐worldness topology associated with the consciousness level (Cacciola et al., 2019). The mentioned general brain network model is affected by single RSN variations linked to changes in the level of consciousness. Additionally, in contrast to the usual description of the interaction between pairs of functional units, we assessed the more global properties of integration, segregation, and centrality that have been suggested to be critical in the emergence of the consciousness phenomena (Tononi & Koch, 2008).

Our analysis indicates that loss of consciousness in patients with DOC is associated with significant changes in the functional connectivity at the RSN level for the three properties studied here. More specifically, severity of consciousness impairments was related to reductions of integration in sensory and cognitively related RSNs, decreases in segregation level for sensory‐related RSNs, and increases of centrality for sensory‐related RSNs. The functional analysis of altered states of consciousness, from the proposed general perspective, reveals a topological reorganization of the large‐scale functional regions which are not described in previous analyses which mainly focused in specific circuits within the RSNs. In summary, we propose a large‐scale (RSNs) functional connectivity model to explore network properties linked to the consciousness phenomena, and we found a reconfiguration of the functional connectivity properties in altered states of consciousness.

The discussion continues arguing about the main findings and implications related to the variations found in the measurements of integration, segregation, and centrality, associated with the consciousness level. It starts by discussing the results over alterations of the integration values from a general perspective, and their link with specific brain circuits in the section, Integration alterations in DOC. Following, section Segregation alterations in DOC considers the variations of the segregation assessments and their relationships with previous findings. Then, in section Centrality alterations in DOC, the changes in the centrality measurements of the large‐scale regions are interpreted. Finally, the discussion indicates some limitations of the presented approach and introduces some perspectives to go further.

### 4.1 Integration alterations in DOC

Integration measurements suggest that RSNs related to awareness are better connected for conscious subjects (Hannawi, Lindquist, Caffo, Sair, & Stevens, 2015). Higher degree values of auditory network, DMN, ECN Left, ECN Right, and visual medial network for HC indicate that, for this population, these RSNs are more connected to other RSNs than in subjects with DOC (Figure 2). This reduction of the degree values in altered states of consciousness could be understood as a reduction of relationships between RSN time‐courses, that is, representative time‐courses are less or not correlated, suggesting an alteration of the functional connectivity structure in this patients. This result corroborates the disruption of external and internal awareness networks (Demertzi et al., 2013) and the decrease in anti‐correlated connectivity previously observed in subjects with DOC (Di Perri, Thibaut, et al., 2014). Also, functional connectivity of salience network was reported as diminished in altered states of consciousness (Guldenmund et al., 2013). This network is usually associated with the orchestration between internal attention and task‐related‐states, and its alterations were linked to consciousness disorders (Heine et al., 2012). In our experiment degree values for salience network support this observation. A more detailed analysis of the integration phenomena can be obtained by studying strength values alterations (Figure 3). These values were also reduced in altered states of consciousness indicating that the amount of information that different time‐courses share is lower for subjects with DOC. This observation confirms the functional disruption associated with the severity of the pathological condition, as was reported for highly detailed networks in distinct consciousness states (Ribeiro de Paula et al., 2017). Further, this reduction could result from a deterioration process of the connectivity between RSNs, which can be an effect of the connectivity drops in small regions (Hannawi et al., 2015). Also, averaged integration measurements, both degree and strength, suggest that preserved levels of consciousness seem to be related to narrow distributions for integration values. In particular, patients with UWS seem to exhibit a larger variety of connectivity values including hyperconnectivity (increment of connectivity) and disconnections, when compared to healthy subjects. Subjects with altered states of consciousness not only reduce the number of connections between RSNs, but also degrade the ones that remain, suggesting a reduction of the synchronization level associated with the communication between networks. Importantly, the measures herein proposed were computed in large spatial regions that contain previously studied areas, such as the thalamo‐cortical circuit (Demertzi et al., 2013). Therefore, breakdowns in integration seem to appear not only for small brain areas, as reported for strength reductions in the connectome computed from EEG (Cacciola et al., 2019), but also for larger functional systems. Similarly to our results, Cacciola et al. (2019) compute integration measurements for subjects with MCS and UWS, but they were also not significant to discriminate between those populations. To conclude, the local integrations measurements corroborates previous findings of connectivity disruptions for patients with DOC, while global integration describes the global integration assessments exhibits a decreasing tendency related to the consciousness level, that is, the conscious subjects seem to be better integrated than patients with MCS, and patients with MCS seem to better integrated than patients with UWS.

### 4.2 Segregation alterations in DOC

High values in clustering coefficient of the DMN seem to be related to the level of synchronization of this network with other RSNs. This result was previously reported in specific awareness circuits involving the DMN (Demertzi et al., 2014). Segregation measurement assessed by clustering coefficient confirms that consciousness could be a phenomenon involving segregated functional units that work in an integrated manner (Tononi, Sporns, & Edelman, 1994). RSNs could be understood as segregated regions that execute specific tasks (Biswal, 2012) but share information in consciousness phenomena (Heine et al., 2012). Sensory and cognitive‐related networks appear to be more clustered for HC. In contrast, the segregation increases for sensorimotor in DOC with no significant differences between MCS and UWS. A similar finding was reported in an experiment with altered states of consciousness and anesthesia (Guldenmund et al., 2017) where an increment of functional connectivity between thalamus and sensorimotor network was found in altered states of consciousness. Altered segregation values in the sensorimotor region, jointly with integration changes, are suggesting a variation in the sensorimotor time‐course behavior, becoming more synchronized with other high‐related RSNs; thus, these variations suggest the configuration of a segregated functional unit. This behavior seems to be a consequence of different scenarios out of the scope of the present study that could be analyzed in future explorations. However, this finding is contrary to the reported by Cacciola et al. (2019) were they reveal an increment of the clustering coefficient in the patients with UWS when compared against MCS, the mentioned difference could be a result of the computation of the clustering coefficient using a binary matrix instead of a weighted connectivity matrix, as in our case. In brief, variations of the segregation measurements in patients with DOC seem to be caused for a reconfiguration of the functional synchronized groups.

### 4.3 Centrality alterations in DOC

Centrality measurements indicate how central a node is in the network. High centrality scores in auditory and sensorimotor networks suggest that these functional units play a central role in patients with altered states of consciousness, revealing a behavior alteration phenomenon even if these regions exhibit a functional connectivity reduction in patients with altered states of consciousness, as was previously reported (Demertzi et al., 2013, 2014; Kirsch et al., 2017). This observation could be further explored to understand the kind of variation induced by DOC that reveals a centrality increment. Similarly, higher scores in sensorimotor network suggest that this network also change its nature, becoming more important in subjects with altered states of consciousness. Interestingly, even if the sensorimotor input‐output loops were reported as not required for consciousness (Tononi & Koch, 2008), the circuits involving these RSN were altered by the pathology (Di Perri, Stender, et al., 2014). A surprising finding is the increment of centrality values for this external awareness network in subjects with DOC, which is not expected due to its associated behavior to sensory stimuli and motor reaction. A similar finding was reported by Cacciola et al. (2019), where an increment of the betweenness centrality in posterior cingulate and visual areas were stated for patients with UWS. However, this finding at RSN level can be a result of a brain reconfiguration in response to not‐conscious stimuli response (Tononi & Koch, 2015). Increases of centrality values of functional connectivity between RSN in altered states of consciousness suggest a modification of their time‐courses nature, becoming more relevant in subjects with DOC. Nevertheless, this new central role of some RSNs would be not suitable for consciousness phenomena, where a sort of equilibrium between segregation and integration is required (Cacciola et al., 2019; Tononi & Koch, 2008, 2015; Tononi et al., 1994). Summarizing, centrality alterations describe a reconfiguration of the relevant functional units in altered consciousness states that seem to be not suitable for the emergence of consciousness.

### 4.4 Limitations and future directions

The analysis developed in this experiment presents some methodological limitations. A potential confounding factor is related to head motion. In order to study this potential bias source, frame‐wise displacements (FWD; Power et al., 2012) were computed for each group to evaluate data quality acquisition. According to FWD, images with major displacements have to be removed for the functional connectome computation of each subject. Average FWD for HC subjects was 0.02 (*SD* = 0.003), for patients with MCS was 0.3 (*SD* = 0.005), and for patients with UWS was 0.04 (*SD* = 0.007). These values indicate that the variation ranges are similar even if the values for MCS and UWS were higher (see Figures S1–S3). Therefore, it is reasonable to assume a small influence of large head motions in the results herein reported. Another potential confounding result is related to the brain gray matter reduction in patients with DOC. Gray matter volume can influence the functional connectome measurements by reducing the amount of voxels which are considered to be in a region, that is, in a RSN, (Table S2). In addition, the proposed analysis was made for functional connectivity between RSNs, that is, for a 10 × 10 matrix representing a broad picture of the brain functionality. This feature limits more specialized analyses as those made to study the topology for larger networks, that is, hubs (Bullmore & Sporns, 2012), and small‐world (Rubinov & Bullmore, 2013; Sporns, 2003). A matrix with more regions would provide a detailed connectivity matrix which exhibits variations in the nodal measurements while the global network assessments remain similar. Indeed, the regions of the detailed matrix can be sorted to arrange the regions into RSN to compare the individual and global measurements. Another consideration to address is the amount of information corresponding to the size of the samples for each population associated to a different state of consciousness, which in this experiment corresponds to 48 patients with DOC (24 MCS and 24 UWS). Additionally, in this study each large‐scale region was represented by an averaged time‐course which explains the neuronal activity of the entire region. This representative time‐course was built from a data‐driven approach, a combination of spatial ICA of neuronal nature (Jafri et al., 2008). Functional connectivity between the representative time‐courses of each RSN was computed by using the lagged distance correlation (Rudas et al., 2014). This approach captures nonlinearities which favors the communication (delayed synchronization) dynamic between RSNs. The window size used in this lagged approach was defined for the HC subjects, taking into account that this fixed size in subjects with DOC might not be suitable if the alterations induced by the pathology affect the synchronization time, that is, cause a communication delay between RSNs. Besides, integration, segregation and centrality measurements herein used permit a broad exploration in a small network. Other graph measurements, like small‐world, rich club, efficiency and shortest paths, have been successfully applied to analyze functional connectivity alterations associated to different pathologies (Rubinov & Sporns, 2010). These measurements provide a better understanding of network topological alterations; however, they require large networks, that is, networks with a large number of nodes. Statistical analysis was based on the family‐wise approach that does not require interpretation of any property. In this work Network‐Based Statistic (Zalesky, Fornito, & Bullmore, 2010; NBS) was not used to identify differences between networks due to the following considerations: (a) the network size; each FNC has 10 nodes that means a maximum of 45 comparisons per network, (b) the power of the contrast ratio suggested in NBS was not suitable. It looks for preserved connections between nodes at different threshold computations which impose a minimum strength in the relationships. These conditions were also indicated by the NBS author, who highlights that the connections comprised the contrast of interest might form components, that is, regions with high power. If they do not form components, or if the extent of the components formed are too small, the NBS is ineffective. This is the case for the 10 × 10 FNC. Finally, in order to get a clear idea about integration, segregation, centrality and topological alterations related to specific areas or circuits in the brain, functional connectivity networks in different scales for the entire brain can be explored. This permits further understanding of time‐courses alterations in sensorimotor and auditory networks to capture the essence of modifications induced by the altered state of consciousness.

## 5 CONCLUSIONS

We use a general model of brain functionality to study its modifications in different states of consciousness. We found that this general model built from large‐scale areas exhibits connectivity alterations induced by the pathology. In particular, we use network measurements to observe modifications of the FNC linked to consciousness level. Our results suggest that the FNC is better integrated and segregated for healthy subjects than for patients with DOC except by the sensorimotor network. Besides, FNC centrality indicates that there exists a role alteration in sensorimotor and auditory networks for patients with DOC where these RSNs become more important.

## CONFLICT OF INTEREST

The authors report no competing interests.

## Data Availability

The data and source code that support the findings of this study are available from the corresponding author upon reasonable request.
