# Supplementary material for: Reconfiguration of large‐scale functional connectivity in patients with disorders of consciousness
Source: Brain Behav. 2019 Nov 26;10(1):e1476. doi: 10.1002/brb3.1476 (PMC6955826; doi:10.1002/brb3.1476)
Supplement: Supplementary file 1 [file BRB3-10-e1476-s001.docx]

**Supporting information: Reconfiguration of large-scale functional connectivity in patients with disorders of consciousness**

#### 1 | Patient demographic information

The Supplementary Table 1. presents the demographic information of the patients with MCS and patients with UWS.

|  |  |  |  |  | **Coma Recovery Scale-Revised** | | | | | | |  |  |
| --- | --- | --- | --- | --- | --- | --- | --- | --- | --- | --- | --- | --- | --- |
| **Alias** | **Sex** | **Age** | **Etiology** | **Days since insult** | **Auditory function** | **Visual function** | **Motor function** | **Oromotor/ Verbal function** | **Commu-nication** | **Arousal** | **Total score** | **MRI/CT** | **EEG** |
| MCS01 | m | 30 | Trauma | 246 | Reproducible movement to command | Fixation | Flexion withdrawal | Vocalization/oral movement | None | With stimulation | 10 | Bifrontal, mid-corpus callosum, bilateral internal capsule & centrum semiovale, R thalamic, mesencephalic, & pontine lesions | BR 13 Hz reactive |
| MCS02 | f | 74 | Sub-arachnoid hemorrhage | 18 | Auditory startle | None | Abnormal posturing | Oral reflex | Intentional | With stimulation | 5 | L parieto-occipital subarachnoid hemorrhage. Leucoencephalopathy in frontal & parietal lobes. Supratentorial hematoma including R thalamus & internal capsule extending to pons | BR 4-5 Hz irregular non-reactive delta |
| MCS03 | m | 52 | Seizure | 20 | Reproducible movement to command | Visual pursuit | Flexion withdrawal | Vocalization/oral movement | Intentional | Without stimulation | 13 | R cortico-subcortical fronto-temporal lesions | BR theta, non-reactive symmetrical |
| MCS04 | f | 43 | Sub-arachnoid hemorrhage | 31 | Auditory startle | Visual pursuit | Flexion withdrawal | Oral reflex | None | Without stimulation | 7 | Anterior interhemispheric subarachnoid hemorrhage | BR theta range |
| MCS05 | m | 65 | Trauma | 28 | Reproducible movement to command | Fixation | Flexion withdrawal | oral reflex | None | With stimulation | 9 | Cerebellar hemorrhage, bilateral parieto-occipital subdural hematoma, bifrontal hematoma (R>L) | BR 5Hz, irregular, non-reactive |
| MCS06 | m | 47 | Trauma | 533 | Reproducible movement to command | Object recogni-tion | Flexion withdrawal | Oral reflex | None | Without stimulation | 13 | Bilateral leucoencephalopathy. Lesions in anterior pons, midbrain & frontal lobe. Secondary hydrocephalus & hippocampal & cerebellar atrophy | BR diffuse theta |

|  |  |  |  |  | **Coma Recovery Scale-Revised** | | | | | | |  |  |
| --- | --- | --- | --- | --- | --- | --- | --- | --- | --- | --- | --- | --- | --- |
| **Alias** | **Sex** | **Age** | **Etiology** | **Days since insult** | **Auditory function** | **Visual function** | **Motor function** | **Oromotor/ Verbal function** | **Commu-nication** | **Arousal** | **Total score** | **MRI/CT** | **EEG** |
| MCS07 | m | 41 | Anoxia | 9900 | Reproducible movement to command | Visual pursuit | Flexion withdrawal | Vocalization/oral movement | None | Without stimulation | 12 | Periventricular leucoencephalopathy. Lesions in bilateral frontoparietal convexity & external capsule. Atrophy of frontal & temporal lobes hippocampi & thalami. Secondary hydrocephalus | BR 6-7 Hz, symmetrical |
| MCS08 | m | 37 | Trauma | 342 | Reproducible movement to command | Object recogni-tion | Localisation to noxious stimulation | Oral reflex | None | Without stimulation | 14 | Lesions in R posterolateral pons, R cerebral peduncles & corpus callosum | NA |
| MCS09 | m | 37 | Trauma | 134 | Reproducible movement to command | Visual pursuit | Flexion withdrawal | Oral reflex | None | Without stimulation | 10 | Mesencephalic, posterior corpus callosum hemorrhage, bi-parietal subarach-noid hemorrhage | BR 6Hz non-reactive, diffuse sporadic delta |
| MCS10 | f | 34 | Trauma | 3034 | Reproducible movement to command | Visual pursuit | Flexion withdrawal | Vocalization/oral movement | None | Without stimulation | 12 | L thalamic contusional lesion. Juxta-cortical lesions in frontal & temporal lobes. Atrophy of L cerebral peduncle & cerebellar vermis | BR 6-7 Hz irregular reactive |
| MCS11 | m | 62 | CVA | 13 | None | Visual pursuit | Flexion withdrawal | Oral reflex | None | With stimulation | 7 | Porencephalic post-ischemic cavity in R sylvian area, pontine & diffuse white matter ischemic lesions, R fronto-parietal & bilateral hippocampal atrophy | BR low amplitude 7 Hz irregular |
| MCS12 | f | 59 | CVA | 21 | Auditory startle | Visual pursuit | Flexion withdrawal | None | None | Without stimulation | 8 | Bifrontal subcortical ischemic lesions Supra-callosal hemorrhage | BR theta, delta range, non-reactive |
| MCS13 | f | 34 | Trauma | 1077 | Reproducible movement to command | Object recogni-tion | Flexion withdrawal | None | Intentional | With stimulation | 12 | Diffuse cortical & subcortical atrophy secondary hydrocephalus | BR theta range, reactive |
| MCS14 | m | 38 | CVA | 1854 | Reproducible movement to command | Visual pursuit | Flexion withdrawal | Vocalization/oral movement | None | With stimulation | 9 | Lesions in L thalamus & posterior limb of L internal capsule, extending to the cerebral peduncle & corpus callosum | BR 5-6 Hz irregular reactive |
|  |  |  |  |  | **Coma Recovery Scale-Revised** | | | | | |  |  |  |
| **Alias** | **Sex** | **Age** | **Etiology** | **Days since insult** | **Auditory function** | **Visual function** | **Motor function** | **Oromotor/ Verbal function** | **Commu-nication** | **Arousal** | **Total score** | **MRI/CT** | **EEG** |
| MCS15 | m | 31 | Trauma | 849 | Reproducible movement to command | Object recogni-tion | Flexion withdrawal | Oral reflex | None | Without stimulation | 13 | Cortico-subcortical atrophy, hypertensive hydrocephalus. Diffuse axonal injury with lesions in right middle cerebellar peduncle, midbrain, right thalamus, semi-oval centers & frontal cortices | BR delta range, low amplitude |
| MCS16 | m | 29 | Trauma & anoxia | 64 | Auditory startle | Visual pursuit | Flexion withdrawal | Oral reflex | None | Without stimulation | 9 | No lesions on CT | BR 7 Hz, irregular non-reactive, symmetrical |
| MCS17 | m | 23 | Trauma | 301 | Auditory startle | Visual pursuit | Flexion withdrawal | Oral reflex | None | Without stimulation | 9 | Fronto-parietal cortical laminar necrosis | BR theta, delta range (R>L) |
| MCS18 | m | 60 | Trauma | 12 | Reproducible movement to command | Object recogni-tion | Automatic motor reaction | Intelligible verbalization | Intentional | With stimulation | 18 | Diffuse axonal injury (R>L). Lesions in R cerebral peduncle, pons & splenium of corpus callosum | BR theta-delta, irregular |
| MCS19 | f | 36 | Anoxia | 460 | Reproducible movement to command | Visual pursuit | Flexion withdrawal | Vocalization/oral movement | Intentional | Without stimulation | 13 | Hippocampal atrophy | BR 8 Hz, symmetrical, reactive |
| MCS20 | f | 68 | Sub-arachnoid hemorrhage | 1383 | Reproducible movement to command | Visual startle | Abnormal posturing | Oral reflex | None | Without stimulation | 8 | Secondary quadri-ventricular hydrocephalus with transepen-dymal resorption & diffuse leukoencephalopathy. Diffuse cerebral atrophy predominant in parietooccipital regions bilate-rally. Subarachnoid hemorrhage stigmata in occipital & temporal lobes (R>L) | posterior BR 5-6 Hz non-reactive, sporadic sharp wave bursts |
| MCS21 | m | 35 | Trauma | 1331 | Reproducible movement to command | None | Flexion withdrawal | Oral reflex | None | Without stimulation | 8 | Cortico-subcortical lesions in bilateral anterior temporal, bi-frontal, R temporo-occipito-parietal & R thalamus, hippocampal atrophy | BR 4 Hz symmetrical, low voltage, non-reactive |
|  |  |  |  |  | **Coma Recovery Scale-Revised** | | | | | |  |  |  |
| **Alias** | **Sex** | **Age** | **Etiology** | **Days since insult** | **Auditory function** | **Visual function** | **Motor function** | **Oromotor/Verbal function** | **Communi-cation** | **Arousal** | **Total score** | **MRI/CT** | **EEG** |
| MCS22 | m | 73 | Trauma | 35 | None | Fixation | None | Oral reflex | None | With stimulation | 4 | Hemorrhagic lesions in R parietal, R temporal,  subdural biparietal hygroma. | BR irregular theta/delta range |
| MCS23 | m | 21 | Trauma | 2078 | Consistent movement to command | None | Flexion withdrawal | Vocalization/oral movement | None | Without stimulation | 10 | L occipital porencephalic cyst. Diffuse cortico-subcortical atrophy & secondary hydrocephalus | BR theta (L>R) diffuse sporadic delta bursts |
| MCS24 | m | 66 | Trauma | 674 | Reproducible movement to command | None | Abnormal posturing | Vocalization/oral movement | None | With stimulation | 7 | Lesions in brainstem, cerebellum, triventricular hydrocephalus | BR non-reactive delta range, diffuse theta dysrhytmia |
| UWS01 | m | 49 | Anoxia | 2889 | Startle reflex | None | Abnormal posturing | Vocalization/oral movement | None | With stimulation | 5 | Anoxic lesions in bilateral basal ganglia. & bi-occipital, diffuse cortico-subcortical atrophy, periventricular leukoencephalopathy | BR irregular very low voltage delta, non-reactive |
| UWS02 | m | 74 | Anoxia | 92 | Auditory startle | None | Abnormal posturing | Oral reflex | None | With stimulation | 4 | Diffuse leucoencephalopathy most pronounced in occipital lobes. Diffuse atrophy in bilateral frontal & parietal lobes, hippocampus & cerebellum. Anoxic lesions in bilateral basal ganglia. Secondary hydrocephalus | BR unstructured non-reactive theta |
| UWS03 | m | 67 | CVA | 43 | Auditory startle | None | Flexion withdrawal | Oral reflex | None | With stimulation | 5 | L medial temporal hematoma extending to L thalamus, posterior internal capsule, lenticular nucleus, insula & Wernicke's area. Diffuse leuco-encephalopathy most pronounced in frontal lobes | BR 6 Hz, non-reactive, symmetric |
|  |  |  |  |  | **Coma Recovery Scale-Revised** | | | | | |  |  |  |
| **Alias** | **Sex** | **Age** | **Etiology** | **Days since insult** | **Auditory function** | **Visual function** | **Motor function** | **Oromotor/Verbal function** | **Communi-cation** | **Arousal** | **Total score** | **MRI/CT** | **EEG** |
| UWS04 | f | 53 | Metabolic (Wernicke's encephalopathy) | 28 | Auditory startle | None | Flexion withdrawal | Oral reflex | None | With stimulation | 5 | Lesions in L thalamic pulvinar, mesencephalon, mammillary bodies, & periaqueductal grey matter | BR 3-4 Hz low voltage, symmetrical |
| UWS05 | f | 63 | Sub-arachnoid hemorrhage | 32 | Auditory startle | Visual startle | None | Oral reflex | None | With stimulation | 5 | Ischemic lesions in cerebellum & brainstem | BR 4-5 Hz irregular non-reactive delta |
| UWS06 | f | 63 | Anoxia | 1210 | Auditory startle | None | Abnormal posturing | Oral reflex | None | With stimulation | 4 | Multiple subcortical lesions in frontoparietal & temporal lobes. Diffuse atrophy in pons, midbrain & thalamus. Quadriventricular hydrocephalus | BR hypovoltage unstructured non-reactive theta |
| UWS07 | m | 29 | Trauma & anoxia | 72 | None | Visual startle | Flexion withdrawal | Oral reflex | None | With stimulation | 6 | Diffuse cortical laminar necrosis, brainstem lesions, diffuse leuco-encephalopathy | BR theta/delta, low voltage |
| UWS08 | f | 74 | CVA | 40 | None | None | Flexion withdrawal | Oral reflex | None | With stimulation | 4 | Ischemic lesions in cerebellum, brainstem, R parietal & occipital cortex, splenium corpus callosum & R thalamus | BR 4-6Hz, irregular, non-reactive |
| UWS09 | m | 58 | Trauma | 24 | None | Visual startle | Flexion withdrawal | None | None | With stimulation | 4 | Bifrontal subdural hematoma, R uncal contusion | BR 5-6Hz, irregular, non-reactive |
| UWS10 | f | 44 | Anoxia | 8 | None | None | Abnormal posturing | None | None | With stimulation | 2 | Lesions in occipital lobes & parieto-temporo-occipital junction. Diffuse cortico-subcortical ischemic lesions | BR diffuse non-reactive low-voltage delta |
| UWS11 | m | 63 | CVA | 30 | Auditory startle | Visual startle | Flexion withdrawal | Vocalization/oral movement | None | Without stimulation | 8 | Bihemispheric ischemic lesions predominantly in posterior parietal & occipital lobes | BR unstructured delta |
| UWS12 | f | 45 | Trauma | 34 | Auditory startle | None | Flexion withdrawal | Oral reflex | None | With stimulation | 5 | R temporo-parietal, occipital & basal ganglia hematoma, corpus callosum lesions | NA |
|  |  |  |  |  | **Coma Recovery Scale-Revised** | | | | | |  |  |  |
| **Alias** | **Sex** | **Age** | **Etiology** | **Days since insult** | **Auditory function** | **Visual function** | **Motor function** | **Oromotor/Verbal function** | **Communi-cation** | **Arousal** | **Total score** | **MRI/CT** | **EEG** |
| UWS13 | f | 27 | Trauma | 806 | Auditory startle | None | Flexion withdrawal | Oral reflex | None | With stimulation | 5 | L fronto-temporo-parietal & brainstem lesions, diffuse leucoencephalopathy, bilateral frontal & temporal pole contusio | NA (craniectomy) |
| UWS14 | m | 87 | Sub-arachnoid hemorrhage | 7 | None | None | Flexion withdrawal | Oral reflex | None | With stimulation | 4 | Bi-frontal subarachnoid hemorrhage | BR diffuse theta |
| UWS15 | f | 41 | Anoxia | 1572 | Auditory startle | None | Abnormal posturing | Oral reflex | None | Without stimulation | 5 | Lesions in corpus callosum, thalami, lenticular nuclei, midbrain, pons & cerebellum. Diffuse atrophy with secondary hydrocephalus | BR unstructured theta |
| UWS16 | m | 44 | CVA | 27 | Auditory startle | None | Abnormal posturing | None | None | Without stimulation | 4 | Ischemic lesions in frontal & temporal lobes, caudate nucleus, thalamus, bilateral insula & hippocampi | BR 6 Hz, irregular, non-reactive, symmetrical |
| UWS17 | m | 14 | Trauma | 257 | Auditory startle | Visual startle | Abnormal posturing | Oral reflex | None | Without stimulation | 6 | Hemorraghic lesions in brainstem, mese-ncephalon, L lenticular nucleus & L fronto-temporal cortex. Diffuse cerebral atrophy & secondary hydrocephalus | BR delta (L>R) |
| UWS18 | m | 25 | Trauma | 486 | Auditory startle | None | Flexion withdrawal | Oral reflex | None | With stimulation | 5 | Lesions in mesencephalon, thalamus (L>R), bilateral orbito-frontal, occipital & temporal poles, diffuse axonal injury, diffuse cortical & subcortical atrophy | BR theta-delta, irregular |
| UWS19 | f | 69 | Anoxia | 50 | Auditory startle | None | Flexion withdrawal | Oral reflex | None | With stimulation | 5 | Bilateral fronto-parieto-temporal leucoencephalopathy including external capsule, caudate nucleus, R thalamus & L insula. Bilateral hippocampal atrophy & hydrocephalus | BR diffuse theta with L lateralized delta |
|  |  |  |  |  | **Coma Recovery Scale-Revised** | | | | | |  |  |  |
| **Alias** | **Sex** | **Age** | **Etiology** | **Days since insult** | **Auditory function** | **Visual function** | **Motor function** | **Oromotor/Verbal function** | **Communi-cation** | **Arousal** | **Total score** | **MRI/CT** | **EEG** |
| UWS20 | f | 49 | CVA | 129 | Auditory startle | None | None | Oral reflex | None | Without stimulation | 4 | Lesions in pons & external capsule. Diffuse leucoencephalopathy | BR diffuse unstructured non-reactive delta |
| UWS21 | m | 36 | Anoxia | 2031 | Auditory startle | None | Abnormal posturing | Vocalization/oral movement | None | Without stimulation | 6 | Bilateral periventricular leucoencephalopathy. Supratentorial cortical & subcortical atrophy including basal ganglia & cerebellum, secondary hydrocephalus | BR 7 Hz, symmetrical |
| UWS22 | m | 34 | Anoxia | 7814 | Auditory startle | Visual startle | Abnormal posturing | Oral reflex | None | With stimulation | 6 | Diffuse cortical & cerebellar atrophy, secondary hydrocephalus | BR 6 Hz, irregular non-reactive, symmetrical |
| UWS23 | m | 33 | Anoxia | 456 | Auditory startle | None | Abnormal posturing | Vocalization | None | Without stimulation | 6 | Ischemic lesions in brainstem, bilateral parieto-occipital cortex, L thalamus, diffuse leucoencephalopathy, diffuse cerebral atrophy with secondary hydrocephalus | BS 6Hz, irregular, non-reactive |
| UWS24 | f | 49 | CVA | 277 | Auditory startle | None | Flexion withdrawal | Vocalization/oral movement | None | With stimulation | 6 | Lesions in brainstem, diffuse leuco-encephalopathy, diffuse cerebral atrophy with secondary hydrocephalus | BR very low voltage symmetrical delta, non-reactive |

MCS: minimally conscious state; UWS: unresponsive wakefulness syndrome; CVA: cerebrovascular accident, BR: basic rhythm

**Supplementary Table 1**. Clinical, structural imaging and electrophysiological data of studied patients. Adapted from Demertzi et al. (Demertzi *et al.*, 2014)

#### 2 | Frame-wise displacement

The Supplementary Fig. 1, 2, and 3 show the frame-wise displacement (FWD) computed as indicated in (Power *et al.*, 2012) during the pre-process of the R-fMRI data. Supplementary Fig. 1 displays FWD of healthy subjects, while Supplementary Fig. 8 and 9 present FWD of patients with MCS and UWS respectively.

**
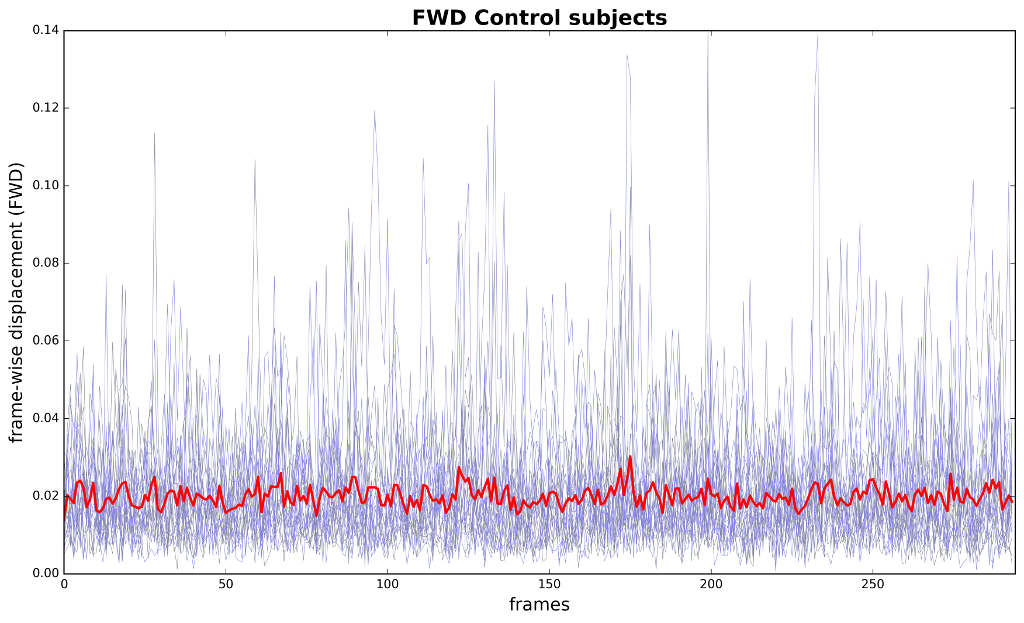
**

**Supplementary Figure 1**. Frame-wise displacement (FWD) computed for healthy subjects. Red line shows the average FWD.

**
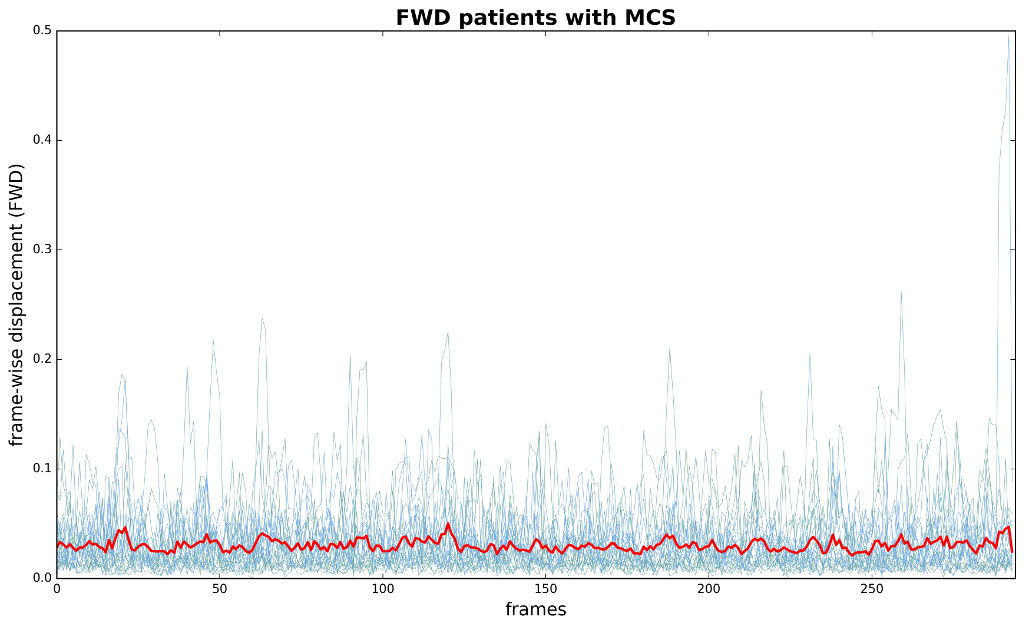
**

**Supplementary Figure 2**. Frame-wise displacement (FWD) computed for patients with Minimally Conscious State (MCS). Red line shows the average FWD.

**
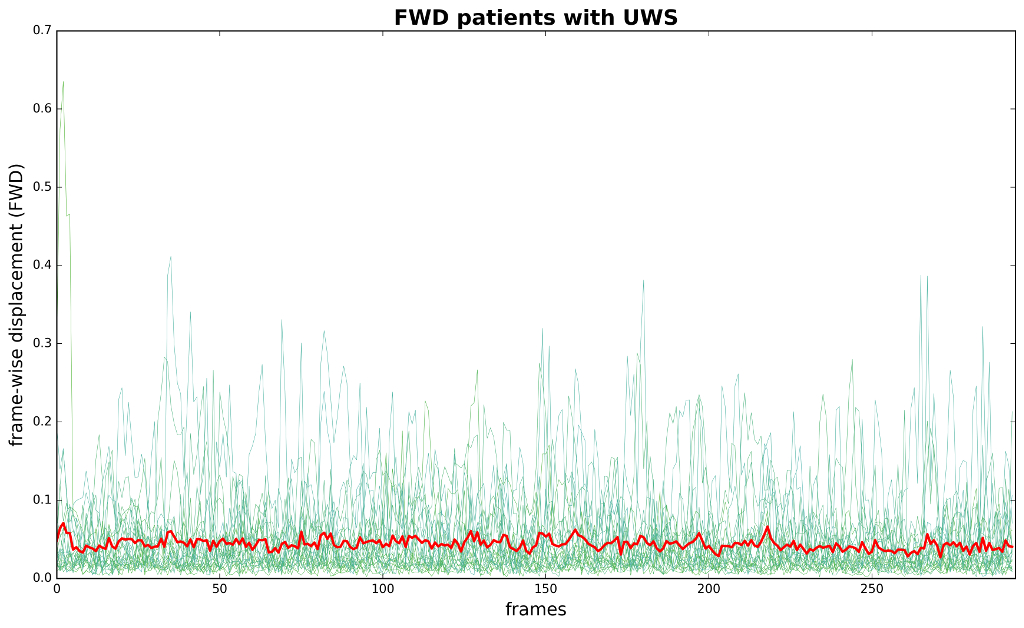
**

**Supplementary Figure 3**. Frame-wise displacement (FWD) computed for patients with Unresponsive Wakefulness Syndrome (UWS). Red line shows the average FWD.

#### 3 | Brain Volume damage versus network measurements

Brain structural damage can influence the graph measurements; the following table presents a relationship between the percentage of brain gray matter damage in contrast to the network measurements used to characterize the functional connectome between RSNs. It is evidence of the direct relationship between brain damage percentage and lower network measurement values.

|  | Brain Damage | Degree | Strength | Clustering Coefficient | Betweenness Centrality | Eigenvector Centrality |
| --- | --- | --- | --- | --- | --- | --- |
|  | Mean (SD) | Mean (SD) | Mean (SD) | Mean (SD) | Mean (SD) | Mean (SD) |
| MCS | 0.10 (0.13) | 2.24 (2.78) | 0.74 (0.83) | 0.12 (0.12) | 0.22 (0.44) | 0.19 (0.08) |
| UWS | 0.23 (0.29) | 1.71 (2.60) | 0.75 (1.20) | 0.11 (0.15) | 0.35 (0.69) | 0.16 (0.08) |
| DOC | 0.16 (0.23) | 1.97 (2.70) | 0.74 (1.03) | 0.12 (0.14) | 0.29 (0.58) | 0.18 (0.08) |

**Supplementary Table 2**. Relation between gray matter damage percentage and network measurements. The percentage of damage was computed as a ratio which take as base the gray matter volume of the healthy subject volunteers (Minimally Conscious State - MCS, Unresponsive Wakefulness Syndrome - UWS, Disorders of Consciousness - DOC)

#### 4 | Extraction and identification of RSNs

This RSNs extraction method was explained in (Demertzi *et al.*, 2014). First, single-subject ICA with 30 components was performed (Ylipaavalniemi and Vigario, 2008) using the infomax algorithm as implemented in the Group-ICA of fMRI toolbox (GIFT: http://icatb.sourceforge.net/). The component images (spatial maps) were calibrated to the raw data so the intensity values were in units of percent signal change (PSC) from the mean (Calhoun *et al.*, 2001). This fit was used to scale the component images into units, which reflect the deviation of the data from the mean, enabling a second level random effects analysis to be performed (Calhoun *et al.*, 2001). The ICs were then matched to the templates representative of the RSNs by means of a goodness-of-fit procedure. This method extends the single-template goodness-of-fit approach (Greicius *et al.*, 2004) by quantifying the absolute PSC average of voxels falling in the template minus the PSC average of voxels outside the template. The templates for each RSN were selected by an expert (author AD) after visual inspection from a set of spatial maps resulting from Group ICA decomposition (30 components running GIFT) performed on 12 independently assessed controls (4 women, mean age = 21 years ± 3, scanned on a 3T MR scanner using a gradient echo-planar sequence of axial slice orientation: 32 slices, voxel-size = 3.4 x 3.4 x 3.0 *mm*^3^, repetition time = 2.460 msec, echo time = 40 msec, flip angle = 90°, field of view = 220 x 220 *mm*^2^ ). These templates were checked by another expert (author AS) for the accuracy of structural labeling. Second, the multiple-template assignation procedure was performed. In order to overcome potentially concurrent IC assignations to the same template, we introduced two physiologic constraints: (i) a template had to be assigned to one of the 30 ICs and (ii) an IC could be labeled as an RSN or not. The first constraint ensured that all templates would be assigned, given that the number of ICs was larger than the number of the templates. The second restriction forced a unique identification of each IC, which overcame the potentially concurrent component assignations. The multiple components labeling with assignation restrictions was formulated as a matching problem:

$${argmax}_{x}=\sum_{i=1}^{N} \sum_{j=1}^{M} X_{i,j}g_{i,j}$$

$$s.t. \sum_{i=1}^{N} X_{i,j}=1, 1\leq j\leq M$$

$$\sum_{j=1}^{M} X_{i,j}=1, 1\leq i\leq N$$

with *M* = 10 the number of different templates, *N* = 30 the number of ICs, *g_i,j_* the goodness of fit between the component *i* and the template *j* and $X_{i,j}\in${0, 1}, an assignation binary variable indicating the match between the template *j* and the IC *i*. Hence, the couple between the template and IC with the highest global goodness of fit (taking into account all templates simultaneously) was eventually selected. The proposed optimization problem was solved by using binary integer programming (Grötschel and Holland, 1985). Third, for the discrimination between “neuronal” and “non-neuronal”, we used a binary classification approach by means of support vector machine (SVM) classifier trained on 19 independently assessed healthy subjects. The feature, which was used for the training of the classifier was the fingerprints obtained from ICA decomposition (n = 30 components). The fingerprint is a feature vector which contains both spatial (i.e., degree of clustering, skewness, kurtosis, spatial entropy) and temporal information (i.e., one-lag autocorrelation, temporal entropy, power of five frequency bands: 0 - 0.008 Hz, 0.008 - 0.02 Hz, 0.02 - 0.05 Hz, 0.05 - 0.1 Hz, and 0.1 - 0.25 Hz) and has been shown to discriminate neuronal from artifactual components (De Martino *et al.*, 2007).

#### 5 | Lagged Distance Correlation

Distance correlation (DC) is a method for measure interaction, accounts for non-linear relationships, between two random variables *X* and *Y* with finite moments in not necessarily equal dimensions (Székely et al., 2007). DC can be defined based on an observed random sample $(X, Y) =\{(X_{k},Y_{k}) | k=1,2,...,n\}$ of the joint distribution of random vectors $X in \mathfrak{R}^{p}$and $Y in \mathfrak{R}^{q}$. Using these samples a transformed distance matrix $A$ can be defined for the sample $X$ as follows:

$$A_{kl}=a_{kl}-\bar{a}_{k\cdot}-\bar{a}_{\cdot l}+\bar{a}_{\cdot\cdot}$$

where $a_{kl}=\left\| X_{k}-X_{l} \right\|$, $\bar{a}_{k\cdot}=\frac{1}{n}\sum_{l=1}^{n} a_{kl}$, $\bar{a}_{\cdot l}=\frac{1}{n}\sum_{k=1}^{n} a_{kl}$, $\bar{a}_{\cdot\cdot}=\frac{1}{n^{2}}\sum_{k,l=1}^{n} \bar{a}_{kl}$for $k,l=1,2,\cdot\cdot\cdot,n$. The matrix $B$ can be similarly defined to characterize distances between samples for $Y$. Note that $A$ and $B$ can be computed independently of *p* and *q*, and both contain information about between sample elements distances in $X$ and $Y$ respectively. The empirical distance between $X$ and $Y$ is defined by

$$V_{n}^{2}(X,Y) = \frac{1}{n^{2}}\sum_{k,l=1}^{n} A_{kl}B_{kl}$$

which is a measure of the distance between the probability distribution of the joint distribution and the product of the marginal distributions, i.e., $V_{n}^{2}\left( X,Y \right)$ quantifies $\left\| f_{X,Y}-f_{X}f_{Y} \right\|$, with $f_{x}$ and $f_{Y}$ the characteristic function of $X$ and $Y$ respectively and $f_{X,Y}$the joint characteristic function (Székely *et al.*, 2007). In contrast to Pearson correlation, $V_{n}^{2}(X,Y)$ is zero if and only if $X$ and $Y$ are independent variables. The empirical DC corresponds to the square root of

$$R_{n}(X,Y)= \frac{V_{n}^{2}(X,Y)}{\sqrt{V_{n}^{2}(X)V_{n}^{2}(Y)}} V_{n}^{2}(X)V_{n}^{2}(Y)>0$$

$$0 V_{n}^{2}\left( X \right)V_{n}^{2}\left( Y \right)=0$$

where $V_{n}^{2}(X) = V_{n}^{2}(X,X)$. The DC corresponds to a normalized version of $V_{n}^{2}(X,Y)$, which takes values between 0 and 1, with zero corresponding to statistical independence between $X$ and $Y$, and 1 total dependency. For the FNC computations, we assumed that two RSN time series $X$ and $Y$ provide the $n$ observations of the joint distribution characteristic of the RSN temporal dynamics. Prior to the DC computations, the RSN time courses were filtered through a bandpass Butterworth filter with cut-off frequencies set at 0.05 Hz and 0.1 Hz. This frequency range was previously used in other studies (Demertzi *et al.*, 2014). Similar to Jafri et al. (Jafri *et al.*, 2008), we used a maximum lagged approach. Lagged Distance Correlation (LDC) is defined by Rudas et al. (Rudas *et al.*, 2014) as:

$$R_{n}^{\Delta}(X,Y)=R_{n}(X,Y^{\Delta})$$

where $Y^{\Delta}$ is the time course circularly shifted ∆ temporal units. We varied ∆ between +6s and -6s in time resolution (TR) units (2s) (Jafri *et al.*, 2008). The maximal DC value for the 7 shifts was defined as the interaction measure between the two RSN time courses. This maximal lagged DC was assessed between all pair-wise valid combinations (both RSNs labeled as “neuronal”) where the number of combinations of 10 RSNs, taken 2 at a time results in $10!/(2!(10-2)!)=45$ possible combinations (Rudas *et al.*, 2014).

#### 6 | Network measurements definition and assessment

| Integration Measurements | | |
| --- | --- | --- |
| Degree | $k_{i}=\sum_{j\in N} a_{ij}$ | Number of links connected to the node (Connection weights are ignored in calculations) (Bullmore and Sporns, 2009; Rubinov and Sporns, 2010). |
| Strength | $k_{i}^{w}=\sum_{j\in N} w_{ij}$ | Sum of weights of links connected to the node (Bullmore and Sporns, 2009; Rubinov and Sporns, 2010). |
| Segregation Measurements | | |
| Number of triangles | $t_{i}^{w}=\frac{1}{2}\sum_{j,h\in N} {(w_{ij}w_{ih}w_{hj})}^{1/3}$ | Number of links connected to the node (Connection weights are ignored in calculations) (Bullmore and Sporns, 2009; Rubinov and Sporns, 2010). |
| Clustering coefficient | $C^{w}=\frac{1}{n}\sum_{i\in N} \frac{2t_{i}^{w}}{k_{i}(k_{i}-1)}$ | Fraction of triangles around a node and is equivalent to the fraction of node’s neighbors that are neighbors of each other (Rubinov and Sporns, 2010; Sporns, 2013). |
| Centrality Measurements | | |
| Betweenness centrality | $b_{i}=\frac{1}{(n-1)(n-2)}\sum_{h,j\in N,h\neq j,h\neq i,j\neq i} \frac{\rho_{hj}(i)}{\rho_{hj}}$ | The fraction of all shortest paths in the network that contain a given node (Rubinov and Sporns, 2010). Where $\rho_{hj}$ is the number of shortest path between *h* and *j*, and $\rho_{hj}(i)$ is the number of shortest path between *h* and *j* that pass through *i*. |
| Eigenvector centrality | $Ax\boldsymbol{=}\lambda x=\frac{1}{\lambda}Ax$  $x_{i}=\frac{1}{\lambda}\sum_{j=1}^{n} a_{ij}x_{j}$ | Self-referential measure of centrality: nodes have high eigenvector centrality if they connect to other nodes that have high eigenvector centrality (Lohmann *et. al.*, 2010). $A$ denotes an $n\times n$ similarity matrix. $x_{i}$is defined as the $i^{th}$entry in the normalized eigenvector belonging to the largest eigenvalue of $A$ |

**Supplementary Table 3**. Brief description of networks measurements used to estimate the integration, segregation and centrality of resting state networks (RSNs) in a network built from functional connectivity between them. $N$ is the set of all nodes in the network, $n$ is the number of nodes, $L$ is the set of all links in the network, and $l$ is the number of links. $(i,j)$ is a link between nodes *i* and *j*. $(i,j)$ are associated with normalized connection weights $0\leq w_{ij}\leq1$. $a_{ij}$ is the connection status between *i* and *j*: $a_{ij}=1$ when link exist and $a_{ij}=0$ otherwise.

The Supplementary Tables 4, 5, 6, 7 and 8 present the statistical values, mean (standard deviation) of the network measurements for the ten RSNs (auditory, cerebellum, default mode network (DMN), executive control left (ECN Left), executive control right (ECN Right), saliency, sensorimotor, visual lateral, visual media and visual occipital networks). They were evaluated for the populations with different consciousness states (Healthy controls - HC, Minimally conscious state - MCS, Unresponsive Wakefulness Syndrome - UWS, and subjects with DOC: union of MSC and UWS). Also, they show the unpaired t-test values, *t* and *p*, with the comparisons among them.

| Network | HC | MCS | UWS | DOC | HC vs. MCS | | | HC vs. UWS | | | HC vs. DOC | | MCS vs. UWS | |
| --- | --- | --- | --- | --- | --- | --- | --- | --- | --- | --- | --- | --- | --- | --- |
|  |  |  |  |  | t | p | t | | p | t | | p | t | p |
| **Auditory** | 5.11 (SD 2.30) | 2.08 (SD 3.20) | 1.60 (SD 2.90) | 1.84 (SD 3.06) | **3.83** | **0.0003** | **4.76** | | **0.00001** | **4.79** | | **0.000008** | 0.54 | 0.59 |
| Cerebellum | 1.67 (SD 2.99) | 1.54 (SD 3.08) | 1.28 (SD 2.75) | 1.41 (SD 2.92) | 0.14 | 0.86 | 0.47 | | 0.63 | 0.361 | | 0.71 | 0.31 | 0.76 |
| **Default Mode Network** | 5.19 (SD 2.11) | 2.08 (SD 3.09) | 1.68 (SD 3.07) | 1.88 (SD 3.09) | **4.14** | **0.0001** | **4.74** | | **0.00001** | **4.9** | | **0.000005** | 0.45 | 0.66 |
| **Executive Control Network Left** | 5.15 (SD 2.29) | 2.75 (SD 3.20) | 2.08 (SD 3.15) | 2.41 (SD 3.19) | **3.04** | **0.003** | **3.96** | | **0.0002** | **3.88** | | **0.0002** | 0.72 | 0.47 |
| **Executive Control Network Right** | 4.74 (SD 2.56) | 2.83 (SD 3.21) | 1.88 (SD 3.06) | 2.35 (SD 3.17) | 2.31 | 0.02 | **3.59** | | **0.0007** | **3.32** | | **0.001** | 1.04 | 0.30 |
| Saliency | 3.19 (SD 3.36) | 1.58 (SD 3.03) | 1.40 (SD 2.70) | 1.49 (SD 2.87) | 1.75 | 0.08 | 2.06 | | 0.04 | 2.29 | | 0.02 | 0.22 | 0.83 |
| Sensorimotor | 2.26 (SD 3.34) | 2.62 (SD 3.17) | 2.16 (SD 3.12) | 2.39 (SD 3.15) | -0.39 | 0.69 | 0.1 | | 0.91 | -0.16 | | 0.87 | 0.51 | 0.61 |
| Visual lateral | 2.41 (SD 3.25) | 2.38 (SD 3.29) | 1.72 (SD 2.92) | 2.04 (SD 3.12) | 0.03 | 0.97 | 0.78 | | 0.43 | 0.47 | | 0.63 | 0.72 | 0.47 |
| **Visual Medial** | 4.93 (SD 2.57) | 2.50 (SD 3.14) | 1.88 (SD 3.12) | 2.18 (SD 3.14) | **2.98** | **0.004** | **3.78** | | **0.0004** | **3.83** | | **0.0002** | 0.68 | 0.49 |
| Visual Occipital | 3.52 (SD 3.35) | 2.04 (SD 3.21) | 1.44 (SD 2.68) | 1.73 (SD 2.97) | 1.57 | 0.12 | 2.41 | | 0.01 | 2.36 | | 0.02 | 0.70 | 0.59 |

**Supplementary Table 4**. Degree values. Mean (Standard Deviation) and the statistic unpaired t-test for each Resting State Network in the different population sets. Resting state networks in bold present significant differences between populations (p < 0.005). (Healthy Controls - HC, Minimally Conscious State - MCS, Unresponsive Wakefulness Syndrome - UWS, Disorders of Consciousness DOC: union of MSC and UWS)

| Network | HC | MCS | UWS | DOC | HC vs. MCS | | | HC vs. UWS | | HC vs. DOC | | MCS vs. UWS | |
| --- | --- | --- | --- | --- | --- | --- | --- | --- | --- | --- | --- | --- | --- |
|  |  |  |  |  | t | p | t | | p | t | p | t | p |
| **Auditory** | 1.72 (SD 0.93) | 0.61 (SD 0.92) | 0.62 (SD 1.22) | 0.62 (SD 1.08) | **4.16** | **0.00001** | **3.59** | | **0.0007** | **4.39** | **0.00003** | -0.03 | 0.98 |
| Cerebellum | 0.48 (SD 0.82) | 0.49 (SD 0.97) | 0.58 (SD 1.35) | 0.54 (SD 1.18) | -0.04 | 0.966 | -0.35 | | 0.731 | -0.23 | 0.815 | -0.28 | 0.78 |
| **Default Mode Network** | 1.72 (SD 0.78) | 0.68 (SD 0.99) | 0.78 (SD 1.47) | 0.73 (SD 1.26) | **4.11** | **0.0001** | **2.86** | | **0.006** | **3.66** | **0.0004** | -0.28 | 0.78 |
| Executive Control Network Left | 1.48 (SD 0.68) | 0.88 (SD 1.02) | 0.97 (SD 1.55) | 0.93 (SD 1.32) | 2.44 | 0.018 | 1.52 | | 0.135 | 2.01 | 0.049 | -0.23 | 0.82 |
| Executive Control Network Right | 1.47 (SD 0.89) | 0.98 (SD 1.10) | 0.96 (SD 1.66) | 0.97 (SD 1.41) | 1.73 | 0.091 | 1.39 | | 0.17 | 1.66 | 0.101 | 0.06 | 0.95 |
| Saliency | 1.06 (SD 1.12) | 0.53 (SD 1.00) | 0.59 (SD 1.19) | 0.56 (SD 1.10) | 1.75 | 0.087 | 1.44 | | 0.157 | 1.86 | 0.067 | -0.19 | 0.85 |
| Sensorimotor | 0.80 (SD 1.18) | 0.94 (SD 1.10) | 0.91 (SD 1.39) | 0.92 (SD 1.26) | -0.42 | 0.674 | -0.32 | | 0.753 | -0.42 | 0.673 | 0.06 | 0.95 |
| Visual lateral | 0.87 (SD 1.19) | 0.83 (SD 1.14) | 0.66 (SD 1.16) | 0.74 (SD 1.15) | 0.13 | 0.896 | 0.62 | | 0.539 | 0.45 | 0.656 | 0.48 | 0.63 |
| **Visual Medial** | 1.66 (SD 0.97) | 0.82 (SD 0.98) | 0.82 (SD 1.44) | 0.82 (SD 1.24) | **3.03** | **0.004** | 2.45 | | 0.018 | **3.03** | **0.003** | 0.002 | 0.98 |
| Visual Occipital | 1.24 (SD 1.21) | 0.62 (SD 0.96) | 0.58 (SD 1.17) | 0.60 (SD 1.07) | 1.97 | 0.054 | 1.96 | | 0.055 | 2.35 | 0.021 | 0.13 | 0.89 |

**Supplementary Table 5.** Strength values. Mean (Standard Deviation) and the statistic unpaired t-test for each Resting State Networks in the different population sets. Resting state networks in bold present significant differences between populations (p < 0.005). (Healthy Controls - HC, Minimally Conscious State - MCS, Unresponsive Wakefulness Syndrome - UWS, Disorders of Consciousness DOC: union of MSC and UWS)

| Network | HC | MCS | UWS | DOC | HC vs. MCS | | | HC vs. UWS | | HC vs. DOC | | MCS vs. UWS | |
| --- | --- | --- | --- | --- | --- | --- | --- | --- | --- | --- | --- | --- | --- |
|  |  |  |  |  | t | p | t | | p | t | p | t | P |
| **Auditory** | 0.29 (SD 0.12) | 0.10 (SD 0.14) | 0.11 (SD 0.18) | 0.10 (SD 0.16) | **4.91** | **0.00001** | **4.11** | | **0.0001** | **4.99** | **0.000004** | -0.17 | 0.87 |
| Cerebellum | 0.08 (SD 0.14) | 0.06 (SD 0.13) | 0.08 (SD 0.18) | 0.07 (SD 0.16) | 0.42 | 0.679 | -0.09 | | 0.932 | 0.16 | 0.873 | -0.44 | 0.66 |
| **Default Mode Network** | 0.30 (SD 0.10) | 0.12 (SD 0.19) | 0.11 (SD 0.19) | 0.11 (SD 0.19) | 4.26 | **0.00009** | **4.54** | | **0.00004** | **4.73** | **0.00001** | 0.24 | 0.81 |
| Executive Control Network Left | 0.26 (SD 0.10) | 0.16 (SD 0.19) | 0.14 (SD 0.20) | 0.15 (SD 0.20) | 2.41 | 0.02 | 2.77 | | 0.008 | 2.76 | 0.007 | 0.37 | 0.72 |
| Executive Control Network Right | 0.25 (SD 0.13) | 0.17 (SD 0.17) | 0.14 (SD 0.21) | 0.15 (SD 0.19) | 1.96 | 0.056 | 2.34 | | 0.023 | 2.38 | 0.02 | 0.54 | 0.59 |
| Saliency | 0.17 (SD 0.17) | 0.09 (SD 0.18) | 0.10 (SD 0.18) | 0.09 (SD 0.18) | 1.54 | 0.13 | 1.45 | | 0.154 | 1.73 | 0.088 | -0.11 | 0.91 |
| Sensorimotor | 0.11 (SD 0.17) | 0.17 (SD 0.20) | 0.15 (SD 0.21) | 0.16 (SD 0.20) | -1.03 | 0.309 | -0.67 | | 0.504 | -0.96 | 0.34 | 0.31 | 0.76 |
| Visual lateral | 0.12 (SD 0.16) | 0.14 (SD 0.19) | 0.11 (SD 0.18) | 0.12 (SD 0.19) | -0.26 | 0.795 | 0.31 | | 0.76 | 0.02 | 0.982 | 0.52 | 0.61 |
| **Visual Medial** | 0.26 (SD 0.14) | 0.15 (SD 0.17) | 0.12 (SD 0.19) | 0.13 (SD 0.18) | 2.59 | 0.013 | **3.07** | | **0.003** | **3.19** | **0.002** | 0.61 | 0.54 |
| Visual Occipital | 0.18 (SD 0.17) | 0.09 (SD 0.14) | 0.09 (SD 0.17) | 0.09 (SD 0.15) | 2.11 | 0.04 | 1.9 | | 0.063 | 2.37 | 0.021 | -0.07 | 0.94 |

**Supplementary Table 6**. Clustering coefficient values. Mean (Standard Deviation) and the statistic unpaired t-test for each Resting State Networks in the different population sets. Resting state networks in bold present significant differences between populations (p < 0.005). (Healthy Controls - HC, Minimally Conscious State - MCS, Unresponsive Wakefulness Syndrome - UWS, Disorders of Consciousness DOC: union of MSC and UWS)

| Network | HC | MCS | UWS | DOC | HC vs. MCS | | HC vs. UWS | | | HC vs. DOC | | | MCS vs. UWS | |
| --- | --- | --- | --- | --- | --- | --- | --- | --- | --- | --- | --- | --- | --- | --- |
|  |  |  |  |  | t | p | t | p | t | | p | t | | p |
| Auditory | 0.67 (SD 1.44) | 0.33 (SD 0.94) | 0.96 (SD 3.58) | 0.65 (SD 2.66) | 0.95 | 0.349 | -0.39 | 0.702 | 0.02 | | 0.981 | -0.81 | | 0.42 |
| Cerebellum | 1.85 (SD 5.60) | 0.08 (SD 0.40) | 0.24 (SD 1.18) | 0.16 (SD 0.89) | 1.51 | 0.137 | 1.38 | 0.173 | 2.04 | | 0.045 | -0.61 | | 0.55 |
| Default Mode Network | 0.59 (SD 1.19) | 0.33 (SD 0.94) | 0.08 (SD 0.39) | 0.20 (SD 0.73) | 0.84 | 0.407 | 2.01 | 0.05 | 1.74 | | 0.087 | 1.21 | | 0.23 |
| Executive Control Network Left | 1.70 (SD 3.80) | 0.33 (SD 1.25) | 0.08 (SD 0.39) | 0.20 (SD 0.93) | 1.66 | 0.104 | 2.09 | 0.042 | 2.59 | | 0.012 | 0.95 | | 0.35 |
| Executive Control Network Right | 0.74 (SD 1.73) | 0.17 (SD 0.55) | 0.00 (SD 0.00) | 0.08 (SD 0.40) | 1.52 | 0.134 | 2.09 | 0.041 | 2.51 | | 0.014 | 1.48 | | 0.15 |
| Saliency | 0.37 (SD 1.22) | 0.25 (SD 1.20) | 0.00 (SD 0.00) | 0.12 (SD 0.85) | 0.35 | 0.73 | 1.49 | 0.143 | 1.02 | | 0.309 | 1.02 | | 0.31 |
| Sensorimotor | 0.30 (SD 1.51) | 0.08 (SD 0.40) | 0.56 (SD 2.74) | 0.33 (SD 1.99) | 0.66 | 0.514 | -0.43 | 0.673 | -0.07 | | 0.946 | 0.83 | | 0.41 |
| Visual lateral | 0.00 (SD 0.00) | 0.08 (SD 0.40) | 0.48 (SD 1.63) | 0.29 (SD 1.21) | -1.06 | 0.293 | -1.5 | 0.139 | -1.21 | | 0.231 | -1.14 | | 0.26 |
| Visual Medial | 0.30 (SD 0.90) | 0.42 (SD 1.41) | 0.32 (SD 1.09) | 0.37 (SD 1.26) | -0.36 | 0.72 | -0.09 | 0.933 | -0.26 | | 0.798 | 0.26 | | 0.79 |
| Visual Occipital | 0.37 (SD 1.22) | 0.08 (SD 0.40) | 0.80 (SD 2.71) | 0.45 (SD 1.99) | 1.08 | 0.286 | -0.73 | 0.468 | -0.18 | | 0.854 | -1.25 | | 0.22 |

**Supplementary Table 7.** Betweenness centrality values. Mean (Standard Deviation) and the statistic unpaired t-test for each Resting State Networks in the different population sets. Resting state networks in bold present significant differences between populations (*p < 0.005*). (Healthy Controls - HC, Minimally Conscious State - MCS, Unresponsive Wakefulness Syndrome - UWS, Disorders of Consciousness DOC: union of MSC and UWS)

| Network | HC | MCS | UWS | DOC | HC vs. MCS | | HC vs. UWS | | | HC vs. DOC | | | MCS vs. UWS | | |
| --- | --- | --- | --- | --- | --- | --- | --- | --- | --- | --- | --- | --- | --- | --- | --- |
|  |  |  |  |  | t | p | t | p | t | | p | t | | p |  |
| **Auditory** | 0.35 (SD 0.14) | 0.46 (SD 0.42) | 0.63 (SD 0.42) | 0.55 (SD 0.43) | -1.29 | 0.203 | **-3.32** | **0.002** | -2.35 | | 0.021 | -1.43 | | 0.16 |  |
| Cerebellum | 0.09 (SD 0.16) | 0.07 (SD 0.14) | 0.07 (SD 0.15) | 0.07 (SD 0.15) | 0.31 | 0.755 | 0.26 | 0.793 | 0.34 | | 0.733 | -0.04 | | 0.96 |  |
| **Default Mode Network** | 0.36 (SD 0.12) | 0.15 (SD 0.21) | 0.09 (SD 0.17) | 0.12 (SD 0.19) | **4.3** | **0.00009** | **6.63** | **0.00000002** | **5.819** | | **0.0000001** | 1.13 | | 0.26 |  |
| **Executive Control Network Left** | 0.32 (SD 0.13) | 0.20 (SD 0.21) | 0.13 (SD 0.18) | 0.16 (SD 0.20) | 2.35 | 0.023 | **4.25** | **0.00009** | **3.54** | | **0.001** | 1.26 | | 0.21 |  |
| **Executive Control Network Right** | 0.32 (SD 0.15) | 0.21 (SD 0.21) | 0.14 (SD 0.21) | 0.18 (SD 0.22) | 2.15 | 0.037 | **3.46** | **0.001** | **3.1** | | **0.003** | 1.08 | | 0.29 |  |
| Saliency | 0.20 (SD 0.21) | 0.09 (SD 0.17) | 0.09 (SD 0.17) | 0.09 (SD 0.17) | 1.99 | 0.052 | 2.02 | 0.049 | 2.42 | | 0.018 | 0.03 | | 0.98 |  |
| Sensorimotor | 0.13 (SD 0.19) | 0.22 (SD 0.23) | 0.14 (SD 0.19) | 0.18 (SD 0.22) | -1.52 | 0.136 | -0.15 | 0.883 | -0.97 | | 0.335 | 1.34 | | 0.19 |  |
| Visual lateral | 0.15 (SD 0.20) | 0.14 (SD 0.19) | 0.13 (SD 0.21) | 0.14 (SD 0.20) | 0.14 | 0.889 | 0.32 | 0.752 | 0.27 | | 0.786 | 0.18 | | 0.86 |  |
| **Visual Medial** | 0.35 (SD 0.17) | 0.20 (SD 0.23) | 0.10 (SD 0.16) | 0.15 (SD 0.20) | 2.61 | 0.012 | **5.38** | **0.000002** | **4.29** | | **0.00005** | 1.75 | | 0.87 |  |
| Visual Occipital | 0.22 (SD 0.20) | 0.13 (SD 0.20) | 0.12 (SD 0.21) | 0.13 (SD 0.21) | 1.5 | 0.139 | 1.76 | 0.084 | 1.91 | | 0.06 | 0.25 | | 0.80 |  |

**Supplementary Table 8.** Eigenvector centrality values. Mean (Standard Deviation) and the statistic unpaired t-test for each Resting State Networks in the different population sets. Resting state networks in bold present significant differences between populations (*p <0 .005*). (Healthy Controls - HC, Minimally Conscious State - MCS, Unresponsive Wakefulness Syndrome - UWS, Disorders of Consciousness DOC: union of MSC and UWS)

###

### Supplementary References

Bullmore, E. and Sporns, O. (2009). Complex brain networks: graph theoretical analysis of structural and functional systems. *Nature reviews. Neuroscience, 10* (3): 186-198. <https://doi.org/10.1038/nrn2575>

Bullmore, E. and Sporns, O. (2012). The economy of brain network organization. *Nature reviews. Neuroscience, 13* (5): 336-349. <https://doi.org/10.1038/nrn3214>

Calhoun, V., Adali, T., Pearlson, G. et al. (2001). Group ICA of functional MRI data: separability, stationarity, and inference. *Proceedings of International Conference on ICA and BSS*. San Diego, CA.

De Martino, F., Gentile, F., Esposito, F. et al. (2007). Classification of fmri independent component using ic-fingerprints and support vector machine classifiers. *NeuroImage, 34* (1): 177-194. <https://doi.org/10.1016/j.neuroimage.2006.08.041>

Demertzi, A., Gómez, F., Crone, J.S. et al. (2014). Multiple fMRI system-level baseline connectivity is disrupted in patients with consciousness alterations. *Cortex, 52*: 35-46. <https://doi.org/10.1016/j.cortex.2013.11.005>

Greicius, M.D., Srivastava, G., Reiss, A.L. et al. (2004). Default-mode network activity distinguishes Alzheimer’s disease from healthy aging: Evidence from functional MRI. *Proceedings of the National Academy of Sciences of United States of America, 101* (13): 4637-4642. <https://doi.org/10.1073/pnas.0308627101>

Grötschel, M. and Holland, O. (1985). Solving matching problems with linear programming. *Mathematical Programming, 33*: 243-259. <https://doi.org/10.1007/BF01584376>

Lohmann, G., Margulies, D.S., Horstmann, A. et al. (2010). Eigenvector centrality mapping for analyzing connectivity patterns in fMRI data of the human brain. *PloS One, 5* (4): e10232. <https://doi.org/10.1371/journal.pone.0010232>

Power, J.D., Barnes, K.A., Snyder, A.Z. et al. (2012). Spurious but systematic correlations in functional connectivity MRI networks arise from subject motion. *NeuroImage, 59* (3): 2142-2154. <https://doi.org/10.1016/j.neuroimage.2011.10.018>

Rubinov, M. and Sporns, O. (2010). Complex network measures of brain connectivity: Uses and interpretations. *NeuroImage, 52* (3): 1059-1069. <https://doi.org/10.1016/j.neuroimage.2009.10.003>

Rudas, J., Guaje, J., Demertzi, A. et al. A method for functional network connectivity using distance correlation. In: *Engineering in Medicine and Biology Society (EMBC), Proceedings of 36th Annual International Conference of the IEEE* (Aug 26-30); Chicago, IL. 2793-2796.

Székely, G.J., Rizzo, M.L., and Bakirov, N.K. (2007). Measuring and testing dependence by correlation of distances. *The Annals of Statistics, 35* (6): 2769-2794. <https://doi.org/10.1214/009053607000000505>

Ylipaavalniemi, J. and Vigario, R. (2008). Analyzing consistency of independent components: An fmri illustration. *NeuroImage, 39* (1): 169-180. <https://doi.org/10.1016/j.neuroimage.2007.08.027>
